# Analysis of the performance of LSTM-DNN models with the consideration of signal complexity in milling processes

**DOI:** 10.1007/s10845-025-02646-w

**Published:** 2025-07-28

**Authors:** Hui Xie, Meng Liu, Ashley Cusack, Guangxian Li, Andrew P. Longstaff, Songling Ding, Wencheng Pan

**Affiliations:** 1 School of Computing & Engineering, The University of Huddersfield, Huddersfield, UK; 2 School of Engineering, RMIT University Bundoora Campus, Bundoora, Australia; 3 School of Mechanical Engineering, Guangxi University, Nanning, China; 4 Department of Finance, Nankai University Binhai College, Tianjin, China

**Keywords:** Deep learning, Time series, DL performance, DL uncertainty, Signal complexity

## Abstract

This paper investigates the impacts of signal complexity on the performance of Deep Learning (DL) models in milling processes, with a particular focus on cutting force due to its critical role in system diagnostics and process monitoring. To compare the Cutter-Workpiece-Engagement (CWE) time-dependent and time-independent processes, signal characterisation was firstly conducted using various feature indicators to quantify signal complexity under different conditions. Multiple DL models were then evaluated through controlled cutting trials to assess how variations in signal complexity affect model performance. The signal analysis revealed that the 14 selected indicators, along with the Recurrent Neural Network–Deep Neural Network (RNN-DNN) models, demonstrated monotonicity as the number of embedded ‘active frequencies’ increased during the “Partial Engagement” or CWE time-dependent process. However, as the depth of the DL models increased, this monotonicity effect diminished. Furthermore, clearer dependencies were observed in the analysis of harmonic prediction performance, particularly with a more pronounced impact on accuracy and uncertainty in the ‘Partial Engagement’ stage compared to the ‘Full Engagement’ stage. In conclusion, the complex performance testing of DL models reveals a significant relationship between signal complexity and model performance. This study underscores the importance of incorporating signal complexity analysis as a critical component in applying DL technology within machining processes, as it provides valuable insights into model performance.

**Graphical abstract:**

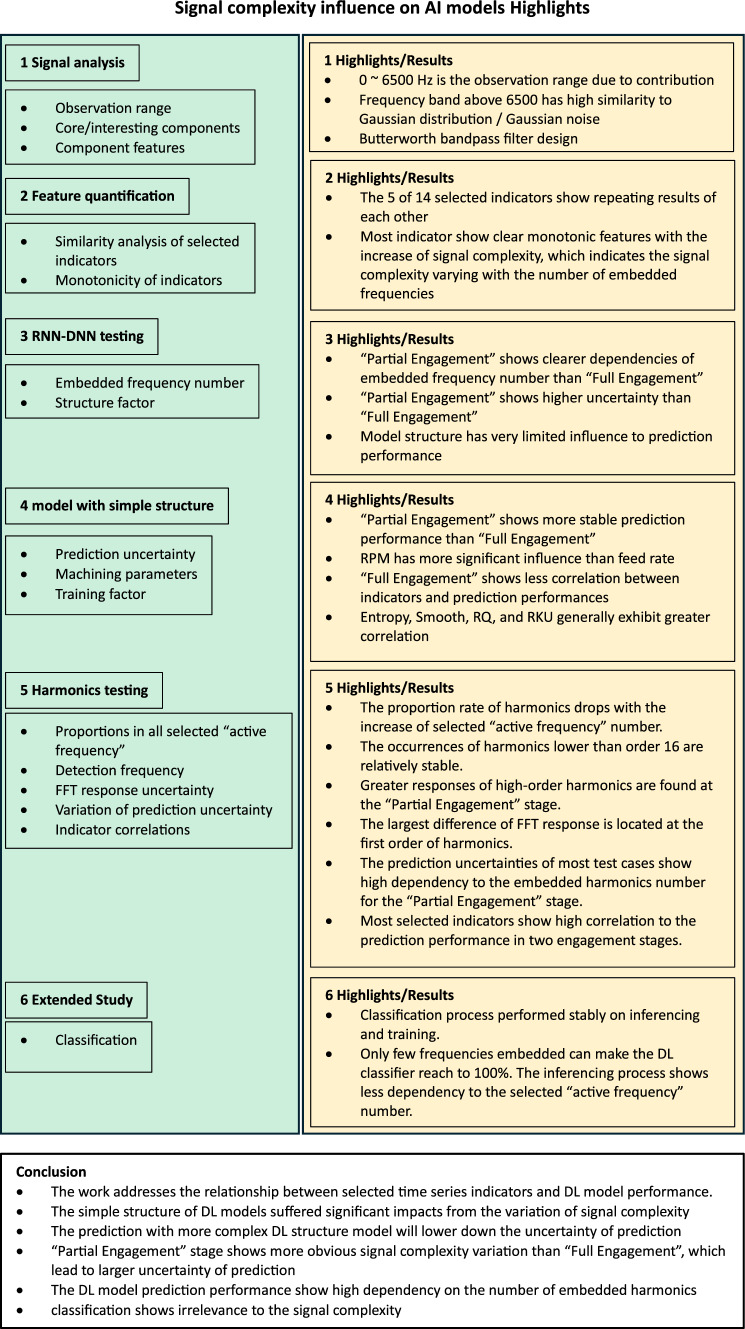

## Introduction

Over the past decade, the applications of artificial intelligence (AI) in machining diagnostics and performance prediction have been intensively studied (Liu et al., 2023a; Moreira et al., 2019; Rajesh et al., 2022; Soori et al., 2023). Meanwhile, the growing use of AI in industry has heightened interest in AI robustness metrology—the measurement of AI systems’ reliability under various conditions (Wang et al., 2023; Wei et al., 2023; Xiang et al., 2023). Although many successful AI-based industrial applications have been reported, quantifying the complexity of training dataset and the quality of modelling data with their influences on AI performance remains a challenge.

The analysis of signal processing techniques for machining diagnostics has been a focal point in recent research, particularly concerning cutting force, acceleration, and acoustic emission signals (Abebe & Gopal, 2023; Das & Bajpai, 2023; Wang et al., 2023; Wu et al., 2023; Xu et al., 2024). Various approaches have been explored to enhance real-time monitoring and predictive accuracy in machining processes. One notable application involved the use of linear filtering, time-domain averaging, and wavelet transformation for real-time tool wear estimation in face milling, as demonstrated by Bhattacharyya (Bhattacharyya et al., 2007). Similarly, Kouguchi (Kouguchi & Yoshioka, 2024) employed frequency response-based motion equations to enable precise in-process monitoring of cutting forces across multiple directions, even under fluctuating and abnormal cutting conditions. This approach highlights the potential of advanced signal decomposition techniques for improving the reliability of cutting force estimation in high-frequency milling environments. Furthermore, acoustic emission signal processing techniques have been widely applied in higher-band frequency feature analysis. For instance, Alzugaray-Franz utilised such techniques for process parameter estimation, while Sakthivel applied them to surface roughness estimation (Alzugaray-Franz et al., 2024; Sakthivel et al., 2024). Collectively, these studies demonstrate the effectiveness of signal processing in machining diagnostics, particularly in tool wear monitoring, force estimation, and surface quality assessment.

The development of Cutter-Workpiece Engagement (CWE) is a critical factor in the study of machining mechanisms, including the identification of cutting force coefficients and the analysis of tool wear mechanisms (Li et al., 2023a; Zhang et al., 2024). Generally, the time-dependent CWE process is considered more “unstable” than its time-independent counterpart. For instance, in the milling of thin-walled cavity parts, instantaneous CWE exhibits significant variation due to changes in structural rigidity (Wang et al., 2021), leading to pronounced fluctuations in the cutting force signal. By contrast, in certain time-independent CWE processes, such as straight micro-end milling, the captured cutting force signal waveform for each cutting cycle demonstrates high repeatability, rendering it relatively “stable” (Wojciechowski et al., 2019). For accurate process prediction and analysis, both scenarios must be thoroughly considered and examined.

For the work of time series data characterisation such as cutting forces, it commonly involves time-domain, phase-domain, and frequency-domain analyses. Ducroux and Zhang utilised low-band signal features and amplitude variations for tool wear identification and surface roughness prediction, monitoring tool wear evolution through cutting force signal analysis (Ducroux et al., 2021; Zhang et al., 2022). Zhang further applied a multi-scale wide convolution residual network (MSW-1D ResNet) to identify tool wear at initial, normal, and severe stages, achieving an accuracy of up to 97.5%. By incorporating both cutting force and acceleration data, studies have demonstrated that surface roughness in machining processes can be accurately predicted, with prediction accuracies ranging from 80 to 95% (Chauhan et al., 2024; Li et al., 2016; Li et al., Mar 2018; Liu et al., 2023b; Wu et al., Apr 2020). The use of acoustic signals is generally focused on high-band signal feature analysis for machining process characterisation and condition monitoring (Gauder et al., 2022; Li et al., 2023b; Uhlmann & Holznagel, 2022). Compared to lower-band features, high-band features capture more distinctive characteristics. For instance, Tansel utilised an Acoustic Emission (AE) sensor to analyse tool wear in micro-end-milling without interference from forced vibration signals typically found below 40 kHz (Tansel et al., 1998). Kakade introduced in-process monitoring methods for chip status and tool wear (Kakade et al., 1994), while AE signals were successfully employed in tool wear prediction using a decision tree technique (Twarddowski et al., 2021).

Beyond feature extraction from signals, some studies have focused on the mechanisms of uncertainty generation and their impact on system analysis. In an early study, a cutting force model for the turning process was developed, accounting for uncertainty factors related to calibration, acquisition, and process influences (Axinte et al., 2001). The results indicated that considering these uncertainties yielded a minimum R^2^ score of 0.95 in force prediction. Hajdu investigated bounded uncertainties in measured Frequency Response Functions (FRFs), successfully validating a method to enhance robust stability (Hajdu & Bachrathy, 2023). Likewise, Ding examined the impact of parameter uncertainty and measurement errors, establishing a dynamic framework for analysing micro-milling stability (Ding et al., 2023). The sources of machine tool uncertainties are varied, including factors such as geometry, acquisition, and process variations (Ma et al., 2023; No et al., 2021a; Singh & Singh, 2020). However, many studies have focused on a limited set of uncertainty factors. For example, Zhuang’s in-depth study of cutter geometry in machining titanium alloys (Ti6Al4V) demonstrated that elastic recovery influences cutting force and surface finish fluctuations (Zhuang et al., 2022). Liu’s research revealed that structural parameters predominantly determine uncertainty in chatter reliability prediction during the turning process (Liu et al., 2016). Furthermore, systematic studies have examined uncertainties in Finite Element Method (FEM) modelling (No et al., 2021b). Rather than analysing individual uncertainty sources, some studies have explored combined uncertainty expressions, as demonstrated in the uncertainty modelling work of Bhattacharyya and Gözü (Bhattacharyya et al., 2021; Gözü & Karpat, 2017).

The application of machine learning (ML) and deep learning (DL) models has yielded substantial advancements in machining diagnostics (Ng et al., 2023; Paletta et al., 2023; Wazirali et al., 2023). Generally, these models undergo fine-tuning and benchmarking, such as adversarial testing, before deployment to optimise performance (Ali, 2023; Li et al., 2023c). However, practical applications reveal that uncertainties can significantly reduce the accuracy of AI models. For example, Das observed notable prediction errors in cutting force simulations generated by an Artificial Neural Network (ANN), with uncertainty effects exceeding 30% and an R^2^ value reaching only 46.41% (Das et al., 2016). Similarly, Xu reported deviations in cutting force peak values of 24.4% and 11.6% using Convolutional Neural Network (CNN)-based and proposed methods, respectively (Xu et al., 2021). Improved AI model performance was demonstrated by Chauhan et al., who achieved average misclassification rates of 0.39% for Support Vector Machines (SVM) and 0.26% for ANN in surface roughness classification (Chauhan et al., 2024).

It is well established that a clean dataset enhances training efficiency and improves AI model robustness. Reducing embedded uncertainties minimises the risk of AI model failure. Consequently, some studies have focused on analysing and improving training dataset quality through various purification methods (Gong et al., 2023; Kim et al., 2023; Sessions & Valtorta, 2006). Budach, for instance, examined the empirical relationships between six data quality dimensions and the performance of classification, regression, and clustering models, highlighting the critical impact of data completeness and feature accuracy on ML algorithms (Budach et al., 2207). In contrast, data consistency and uniqueness were found to have a comparatively lesser influence. The significance of data quality has also been addressed in research aimed at enhancing training datasets through different methodologies (Dai et al., 2018; Heitz et al., 2023; Kim et al., 2023).

Rather than defining uncertainty through completeness, feature accuracy, and target accuracy, some studies have employed complexity algorithms. For example, Chen introduced permutation entropy and transition entropy, along with a novel Permutation Weighted Statistical Transition Entropy (PWSTE), for time-series characterisation (Chen et al., 2023). PWSTE demonstrated superior sensitivity and effectiveness in identifying time-series data patterns. Similarly, Baldán developed a feature set incorporating 55 traditional vector-based classification algorithms (Baldán & Benítez, 2023). Importance analysis revealed that approximation entropy and simple entropy were the most influential indicators, with an average ratio of approximately 0.4, while the period index remained at zero for both indicators.

Given the critical influence of data quality and complexity on the performance of deep DL models, it is essential to examine their specific effects in practical applications. However, current research primarily focuses on enhancing data preprocessing techniques and optimizing model architectures, often overlooking the intrinsic characteristics of input signals. To address this gap, this study aims to systematically investigate the impact of signal complexity on DL model performance in milling processes, with a particular emphasis on cutting force signals. By analysing signal complexity through various feature indicators and evaluating DL models across different complexity levels, this research seeks to elucidate how variations in signal characteristics—particularly those related to the frequency spectrum and harmonics—affect prediction accuracy, stability, and performance in forecasting, classification, and clustering tasks. Furthermore, this study will examine both time-dependent and time-independent CWE processes by segmenting a slotting process into two distinct phases: the “Partial Engagement” stage and the "Full Engagement" stage. Consequently, the findings will provide insights into the relationship between signal complexity and DL model performance, forming a foundation for the design and selection of DL models tailored to specific signal characteristics.

## Experiment setup

### Experiment platform

**Fig. 1:**
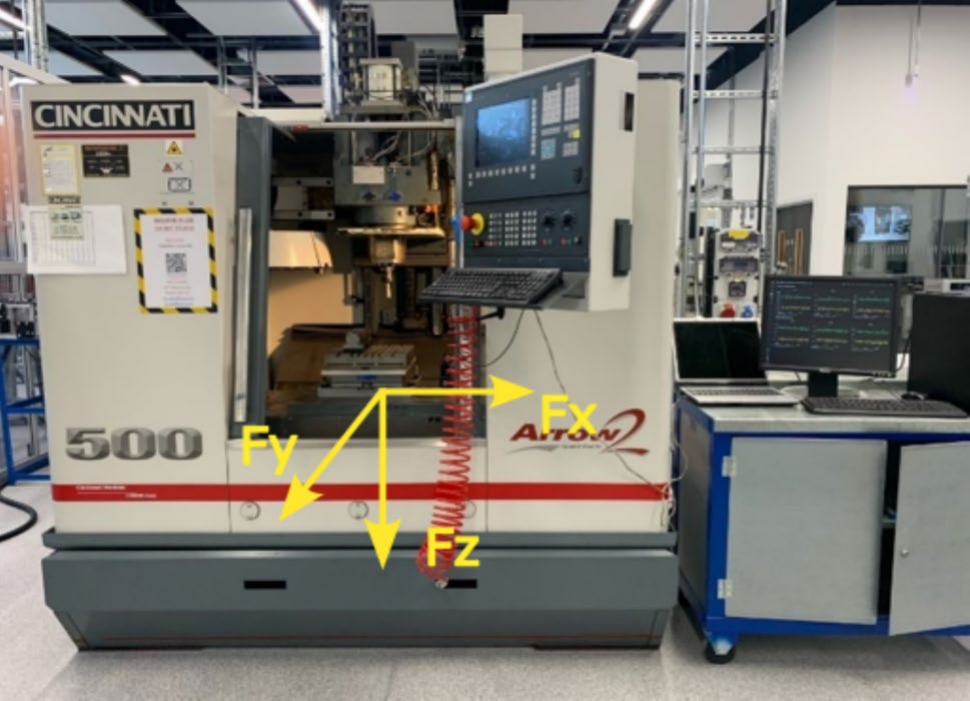
CINCINNATI arrow 2–500 CNC with flooding cooling system with setup of Kistler Dynamometer 9255B, 5017Amplifier and DAQ 5697A

The milling experiments were conducted using a Cincinnati Arrow2 500 CNC, which served as the machining platform. This system is a three-axis CNC milling machine, equipped with a spindle power of 20 kW and capable of exerting torque loads of 10 k Nm along the X and Y axes, while the Z axis supports a torque of 20 k Nm. The spindle operates within a speed range of 0–10,000 RPM, and the machine accommodates a maximum machining volume of 700 mm × 700 mm × 700 mm. Following calibration, the average surface roughness achieved in the slotting process was recorded at less than 10 µm. A Kistler Dynamometer (9255B) was mounted as the working table (Fig. 1) and integrated with a 5017 Amplifier and a DAQ (5697A) system. Each cutting process was monitored and visualised using Dynoware software, developed by Kistler, which offers a maximum sampling frequency of approximately 70 kHz per channel.

The workpiece material used in the study was Al5083, with individual blocks measuring 30 mm × 30 mm × 20 mm. Further specifications regarding the cutter and workpiece are provided in Table 1. The workpiece material used in the experiments was Al5083, with each individual block measuring 30 mm × 30 mm × 20 mm. Further details regarding the cutter and workpiece specifications are provided in Table 1.

**Table 1:** Machining parameters

Test group	Trail no	RPM	DOC (mm)	Feed (mm/min)	Feed (mm/rev)	Engagement (%)	Tool radius (mm)
1	1, 2, 3, 4	3000	1	400	0.1333	100	6
2	5, 6, 7, 8	3000	1	750	0.2500	100	6
3	9, 10, 11, 12	5000	1	800	0.16	100	6
4	13, 14, 15, 16	5000	1	1500	0.3	100	6
Tool			Diameter	12 mm	Applied Workpiece	Aluminium alloys ^a^	
2P170-1200-NA H10F			Helix angle	25°	Tool Material	Solid Carbide	
			Radial rake angle	17°	Depth of cut maximum	48 mm	
			Axis rake angle	1°	Flute NO	2	
			Corner radius	0.15 mm			
			Functional length	99 mm			

^a^ISO N—Non-ferrous metals are softer metals (Bhattacharyya et al., 2021; No et al., 2021b)

### Experiment design

To ensure the machining process remained reproducible, each set of machining parameters was tested four times. As a result, four separate test groups were established, comprising a total of 16 cutting trials. As detailed in Table 1, each test group was allocated a distinct set of machining parameters, which were consistently applied across the four trials within that group. Throughout the experiments, flood cooling was utilised to reduce heat generation and minimise thermal deformation during the milling of Al5083. Tool wear was assessed using an ALICONA optical measurement system at both the start and end of each trial. Upon completion of all 16 tests, the recorded tool nose wear was 0.11 mm, remaining well within the tool failure limit of 0.3 mm, as defined by ISO 8688–2:1989.

### Defining cutter-workpiece-engagement (CWE) stages

Equation (1) is the parametric expression of the cutter nose travelling trajectory in the feed direction.

1 $$ x_{t} = r_{tool} \sin \left( {\frac{2\pi *n}{{60}}t} \right) + f_{t} t $$

As this equation is transcendental, it possesses an infinite number of possible solutions. To estimate the “entrance angle” and “exiting angle,” the Newton–Raphson method is considered an optimal approach. Considering fundamental machining principles, two theoretical boundary conditions can be established: ${x}_{t}=0$ and $0\le \omega t\le \pi$. Consequently, the cutter’s entry and exit times can be determined using Eq. (2).

2 $$ t_{n + 1} = t_{n} - \frac{{r_{tool} \sin \left( {\omega t_{n} } \right) + f_{t} t_{n} }}{{\omega r_{tool} \cos \left( {\omega t_{n} } \right) + f_{t} }},\omega = \frac{2\pi *n}{{60}} $$

#### CWE process type I—“Partial engagement”

**Fig. 2:**
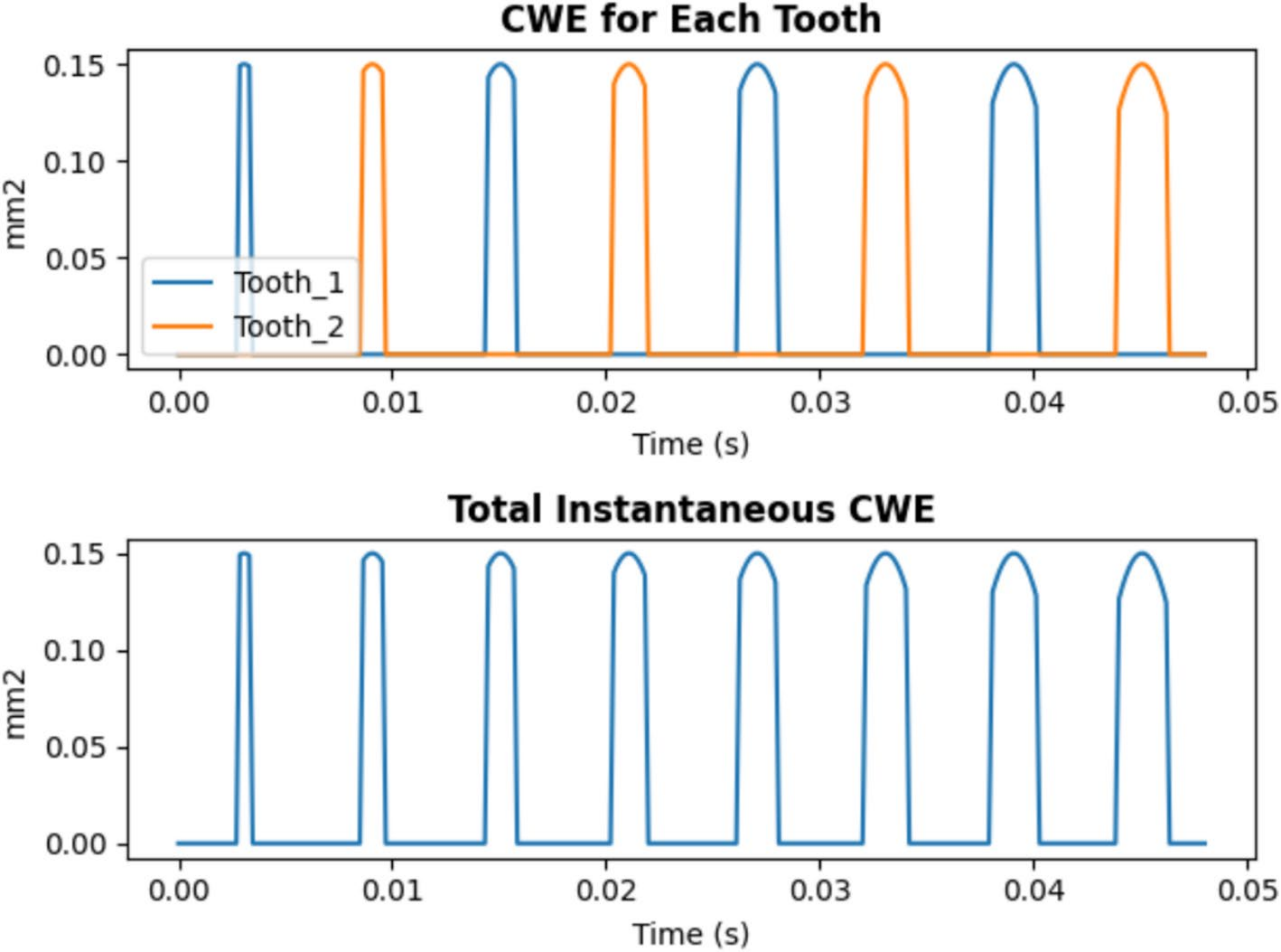
Theoretical CWE of individual flute identification and combination of the “Partial Engagement” stage

For each tooth, the theoretical contact duration per cycle is anticipated to be less than π/ω seconds. To maintain computational precision, the number of iterations for Eq. (2) is fixed at 100. Examples illustrating the “entering angle” and “exiting angle” under varying machining parameters are presented in Fig. 2.

#### CWE process type II—“Full engagement”

This stage is defined as “Full Engagement” when the theoretical contact duration per cycle reaches π/ω seconds. However, in practical applications, this duration fluctuates over time due to the residual uncertainties inherent in the machine tool system.

## Characterisation

### Determination of low band boundary

For the cutting force signal, not all the frequencies are valuable to research. It is necessary to select an observation range for keeping the effective frequencies of the signal.

**Fig. 3:**
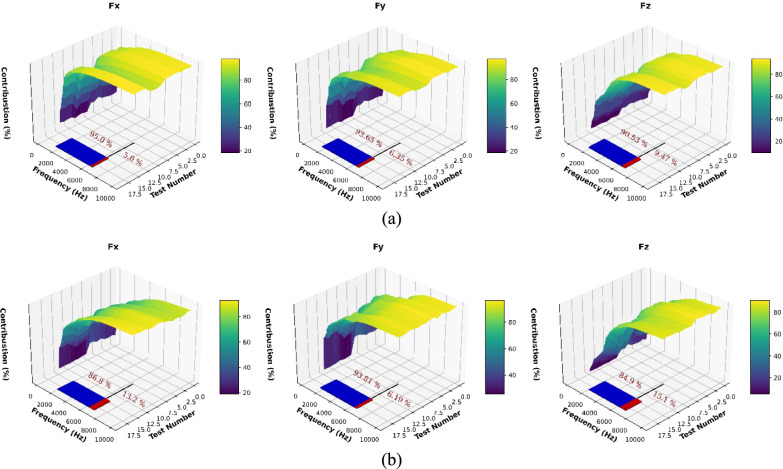
Accumulated contribution analysis (**a**. the “Partial Engagement”, **b**. the “Full Engagement”)

Figure 3 shows the frequency contribution of cutting force for the signals of four the cutting trials selected from each test group. The preset sampling frequency was 70 kHz. In this paper, frequency contribution will be introduced as a method to evaluate which frequency ranges of the cutting force components provide the most significant contribution during the cutting process. The calculation for the frequency contribution is as follows: firstly, the signal is transformed using Fast Fourier Transform (FFT), which converts the time-domain signal into its frequency-domain representation and then remove the direct current components (Li et al., 2020). The resulting frequency components are normalized to obtain their relative magnitudes. To calculate the contribution of specific frequency ranges to the overall signal, the frequency components are grouped into intervals (e.g., 0–100 Hz, 100–200 Hz, etc.). For each interval, the sum of the normalized coefficients within that frequency range is computed. The contribution of that interval is then calculated as the ratio of this sum to the total sum of all coefficients, expressed as a percentage. This ratio represents how much energy the signal has in that specific frequency range relative to the entire spectrum.

The formula for calculating the contribution $CC({f}_{low},{f}_{high})$ within a frequency range $[{f}_{low},{f}_{high}]$ is:

3 $$ CC\left( {f_{low}, f_{high} } \right) = \frac{{\mathop \sum \nolimits_{{f_{i} \in \left[ {f_{low},f_{high} } \right]}} c_{i} }}{{\mathop \sum \nolimits_{{f_{i} }} c_{i} }} \times 100\% $$

where ${c}_{i}$ are the normalized FFT coefficients corresponding to the frequency ${f}_{i}$. This formula quantifies the percentage of the total signal energy that resides within the specified frequency range.

**Fig. 4:**
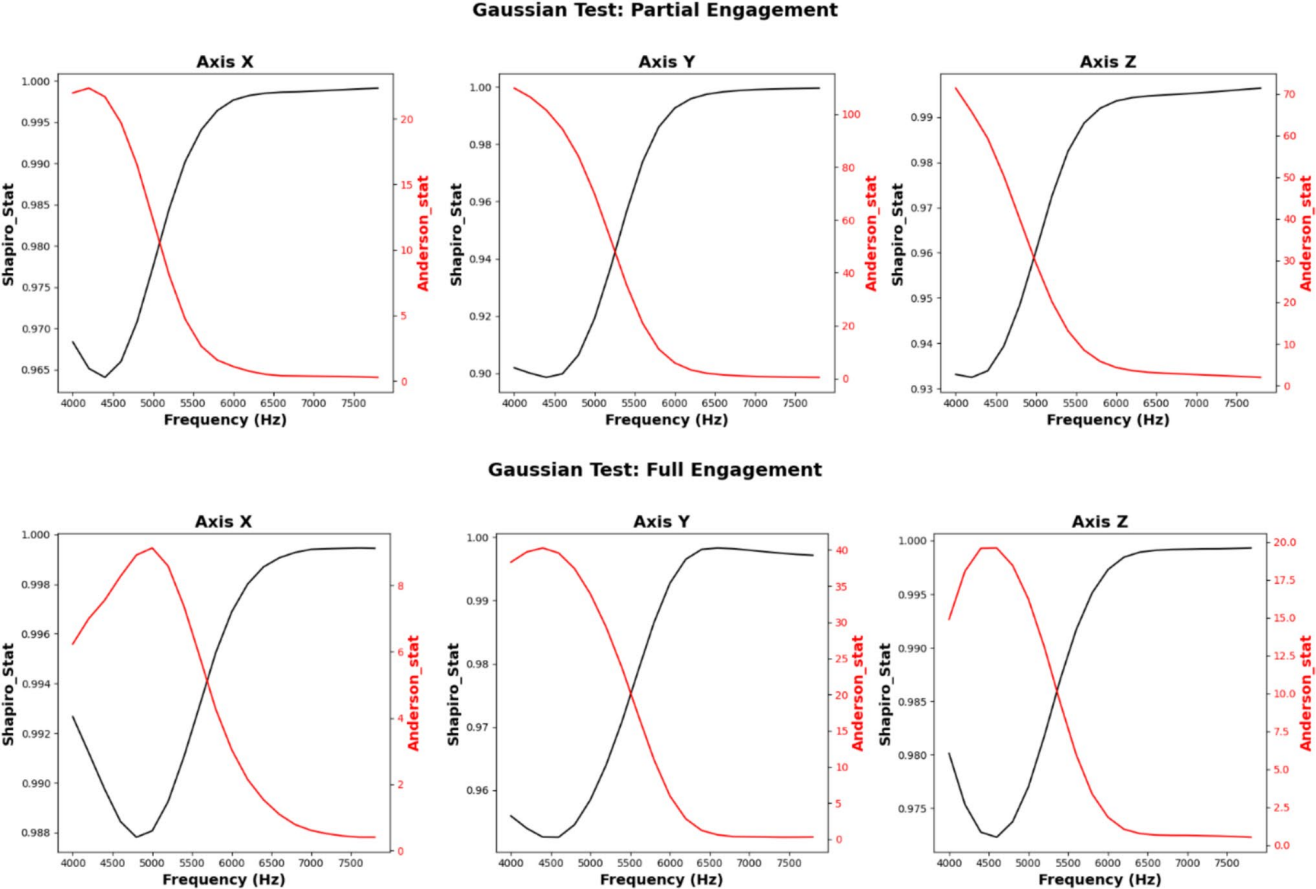
Gaussian testing on cutting force higher band signals

From the results, the gradient of $\mathrm{CC}$ curves slow after 3000 Hz. The average value of $\mathrm{CC}$ at 3000 Hz is higher than 70%. However, in this study, to ensure there is no significant compromise at the signal fidelity after filtering, the boundary of observed frequency is set to be 5000 Hz. Figure 3 shows the accumulated contribution before 6500 Hz reached greater than 90.53% during partial engagement cutting stage. During the full engagement cutting stage, the accumulated contribution before 6500 Hz was greater than 84.9% (average). That means the main contribution is from the cutting force signal under 6500 Hz. Regarding the distribution, Fig. 4 shows an example of the statistical analysis based on the measurements of the **Test Group 4**, **Cutting Trial 13**. As well as the analysis of other test, the range of signal components at higher band is far smaller than the raw signal, and the signal samples are normally distributed with very little bias by comparing with raw signal.

**Fig. 5:**
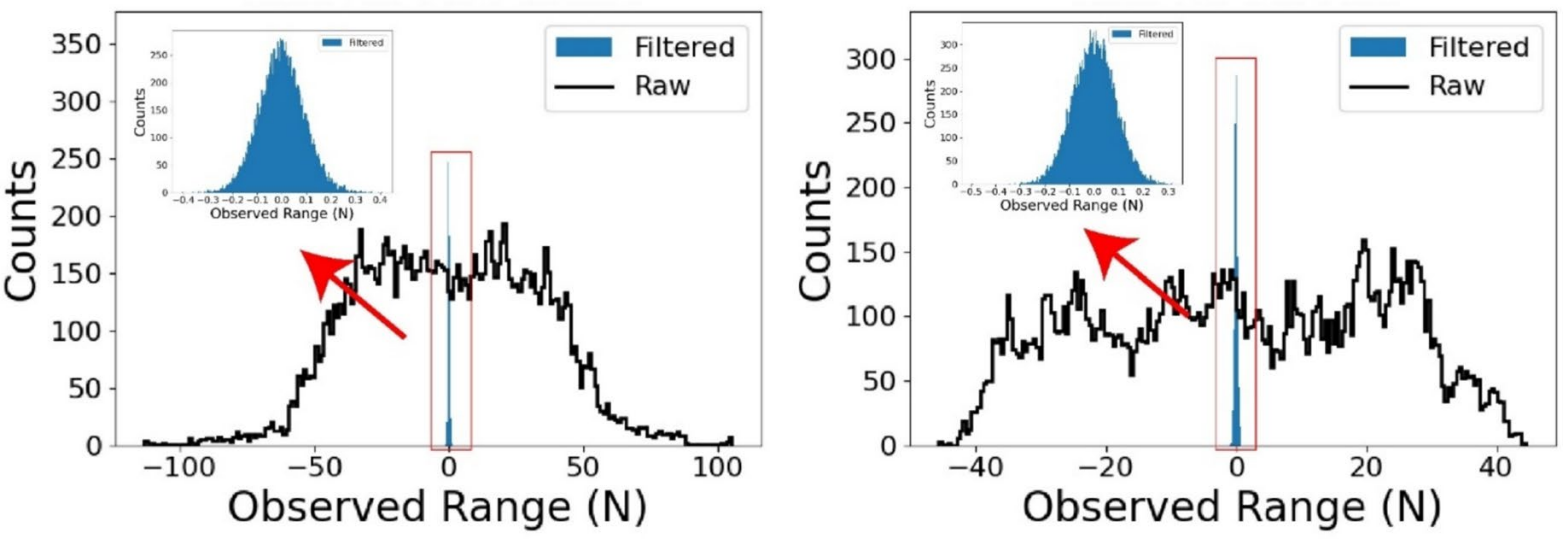
Distribution analysis of filtered signals

For further analysis, Fig. 4 presents the results of the Shapiro–Wilk and Anderson–Darling tests, which assess the normality of the signal components. The Shapiro–Wilk test results indicate values close to 1, suggesting that the signal components in the higher frequency bands follow a normal distribution with minimal bias. This implies that the distribution of these components is relatively consistent with Gaussian behaviour. Additionally, the Anderson–Darling test supports this observation, further confirming the normality of the signal at higher frequencies. Figure 5 illustrates a comparison between the raw measurements and the filtered signals obtained using a low-pass filter, where the cut-off frequency was set at 7 kHz. The filtered signal shows a strong resemblance to a Gaussian distribution, indicating that most signal components beyond 7 kHz can be classified as noise. This behaviour reinforces the idea that these higher frequency components do not carry valuable information for the analysis of the milling process. By integrating these observations, it can be concluded that selecting signal components below 7 kHz will preserve signal fidelity while excluding unnecessary noise. As a result, signal components above 7 kHz are considered noise and can be disregarded in further processing without compromising the quality of the data.

### Signal features analysis

#### The “active frequency” of cutting force

In the context of the FFT, an “active frequency” refers to frequency components within a signal that exhibit significantly higher amplitudes compared to others. These are the dominant frequencies that contribute most substantially to the signal’s composition. In an FFT result—which transforms a time-domain signal into its frequency-domain representation—each frequency bin corresponds to a specific frequency, with its amplitude reflecting the signal’s strength at that frequency. Active frequencies are typically identified by setting a threshold, either absolute or relative to the signal’s overall power or noise level, such that only frequencies with amplitudes exceeding this threshold are considered “active”. These frequencies are often of particular interest in signal analysis, as they represent the most energetically significant components. In general, the complexity of the raw signal is determined by the number of active frequencies, which can direct influence the strategy of signal processing.

#### Comparison between raw signal and filtered signal

**Fig. 6:**
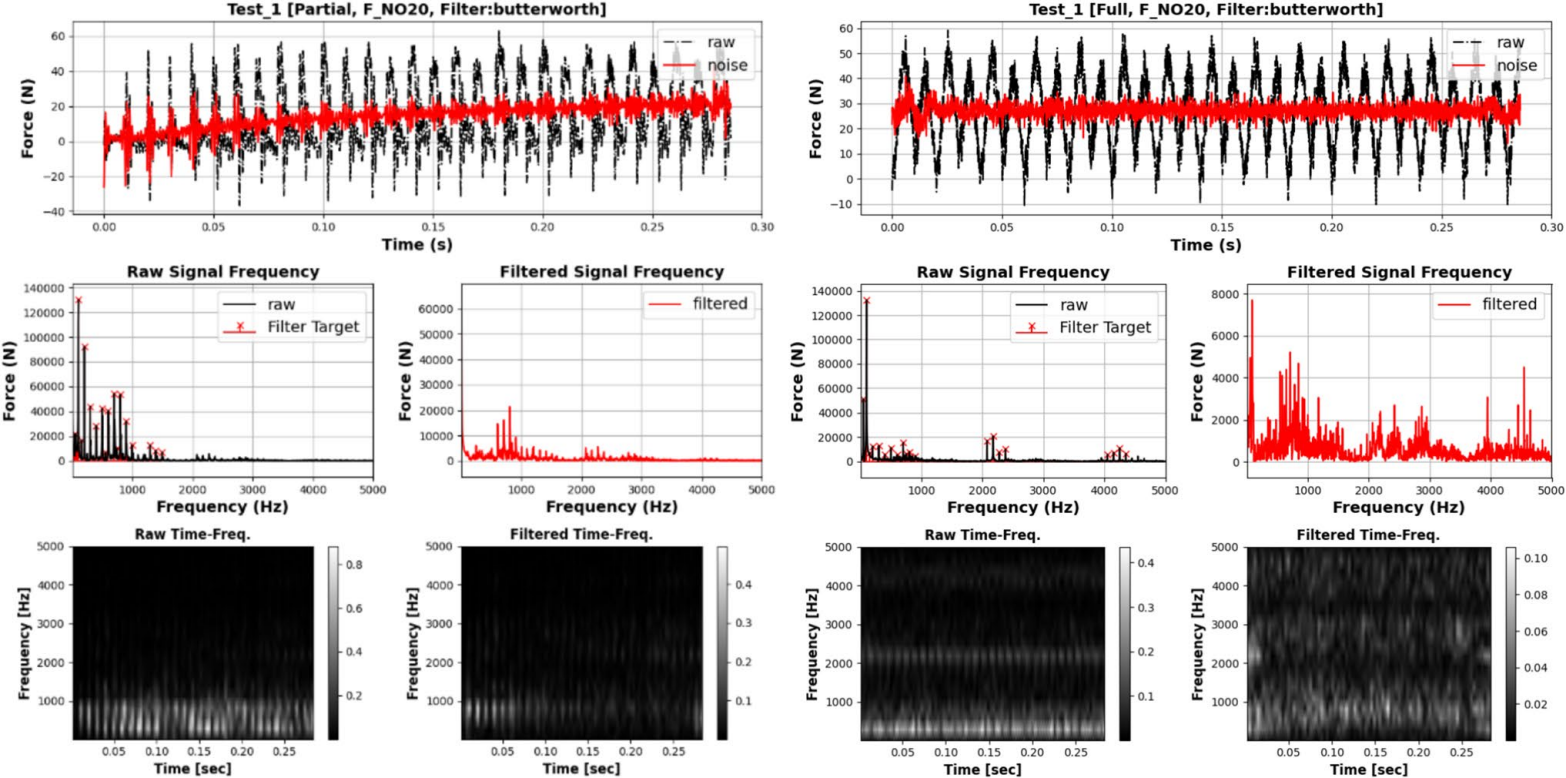
Signal filtering example

The primary objective of this study is to investigate how increasing signal complexity affects the accuracy of cutting force predictions. To achieve this, digital filters are employed to control the number of active frequencies. The filtered signals, containing specific active frequencies, are then used to compute signal characteristics using selected indicators such as Shannon Entropy. For instance, if the number of active frequencies is set to 20 for analysis, the 20 frequencies with the highest absolute FFT coefficients are retained, while the remaining frequencies are filtered out. The observation range for the number of active frequencies spans from 10 to 120, with incremental steps of 2. The Butterworth filter is chosen for its computational efficiency, flat passband, and moderate roll-off characteristics. Figure 6 presents a comparison between the raw and filtered signals from the **Test Group 1**, **Cutting Trial 1** measurement, reflecting similar phenomena observed in other tests.

Firstly, the application of Butterworth digital filters efficiently removed Tooth-Passing Frequency (TPF) and its harmonics of the cutting force signal of the “Partial Engagement” stage and the “Full Engagement” stage. However, the comparison between raw signal and filtered signal indicates that with the same setup of filtering conditions, the filtered signal of the “Partial Engagement” stage still contains significant responses at multiple bands, while the filtered signal of the “Full Engagement” stage is relatively “evener” or “cleaner”. One of the possible explains is that due to the variation of CWE duration, the cutters were suffering dynamic balancing processes at the “Partial Engagement” stages. This situation inevitably brings “additional” frequencies. The captured cutting force signals at the “Full Engagement” stage has much lower fluctuations of the CWE duration. Then, their spectrums were cleaner. And the corresponding filtered signals have higher linearity. The time–frequency heat map of filtered signals strongly supports this conclusion. From the image, the CWE duration varied at each cutting cycle during the “Partial Engagement” stage, which lead to the obvious variations of frequency layout. The more regular frequency layout was observed at the “Full Engagement” stage. Overall, the characteristics of two tested CWE stages indicate that the prediction of cutting force signal at the “Partial Engagement” stage is harder due to larger active frequency number.

### Signal characterisation

To quantify the influence of signal complexity on the performance of deep learning models, this study introduces 14 time-series feature indicators to characterise signals with varying numbers of active frequencies. The indices, names, and capability descriptions of these factors are listed in Table 2. Generally, increasing the number of band-passed frequencies leads to a more complex signal with less distinct features. The variation in signal complexity, which can be quantified using the selected indicators, is expected to be observed during this process. The performance testing results of the deep learning models using the corresponding filtered signals can then be directly correlated with the variation in signal complexity.

**Table 2:** Signal characteristics indicators

NO	Names	Descriptions
F1	Shannon entropy	Shannon Entropy can detect nonlinearity aspects in model series, contributing to a more reliable explanation regarding the nonlinear dynamics of different points of analysis, which in turn enhances the comprehension of the nature of complex systems characterised by complexity and nonequilibrium
F2	Relative entropy	A measure of how one probability distribution diverges from a second, giving an expected probability distribution. This case takes 2 as logarithmic base
F3	Cross entropy	Cross entropy measures the difference between two probability distributions, typically used in machine learning to evaluate the performance of classification models. It quantifies the “cost” of predicting an outcome, with higher values indicating larger discrepancies between predicted and actual distributions, guiding model adjustments to minimise errors
F4	Smoothness	Smoothness if calculated by differentiating a time series (i.e., considering the differences between consecutive observations) can help in identifying and removing patterns to analyse the data’s noise component
F5	Hurst exponents	The Hurst exponent is a statistical measure used to analyse the long-term memory of time series data, particularly in the fields of finance, geology, and hydrology
F6	Dynamic time wrapping	Dynamic Time Warping (DTW) is an algorithm for measuring similarity between two temporal sequences that may vary in speed. It aligns sequences by warping the time axis to minimise differences, making it useful in speech recognition, gesture analysis, and other applications where time-based data comparisons are needed
F7	Volatility	Volatility is the degree of variation in the series over a period, usually quantified by the standard deviation or variance of the returns or changes in the series
F8	Self-correlation	Cross-correlation is an algorithm for finding repeating patterns, such as the presence of a periodic signal obscured by noise or identifying the time delay between two signals
F9	Cross-correlation	Cross-correlation is a statistical measure that quantifies the degree to which two time series are correlated, indicating the similarity and temporal relationship between the series at different time lags
F10	Root-mean -square deviation	A measure of the average deviation of the time series from its mean value
F11	Maximum height of profile	the difference between the highest peak and the lowest valley
F12	Skewness	Skewness is a measure of the asymmetry of a distribution. It indicates whether the data is skewed to the left, right or symmetric
F13	Kurtosis	a measure of the “tailedness” of a probability distribution. It quantifies the shape of the distribution; specifically, how heavy the tails are compared to a normal distribution
F14	Noise zone	The differences between measurements and filtered signals

#### Signal indicator cross-comparative analysis

Although the selected factors characterise sequential data through distinct mechanisms, some exhibit a high degree of similarity. From a numerical perspective, analysing these similarities is essential to prevent redundant assessments and to maintain the uniqueness of each factor.

**Fig. 7:**
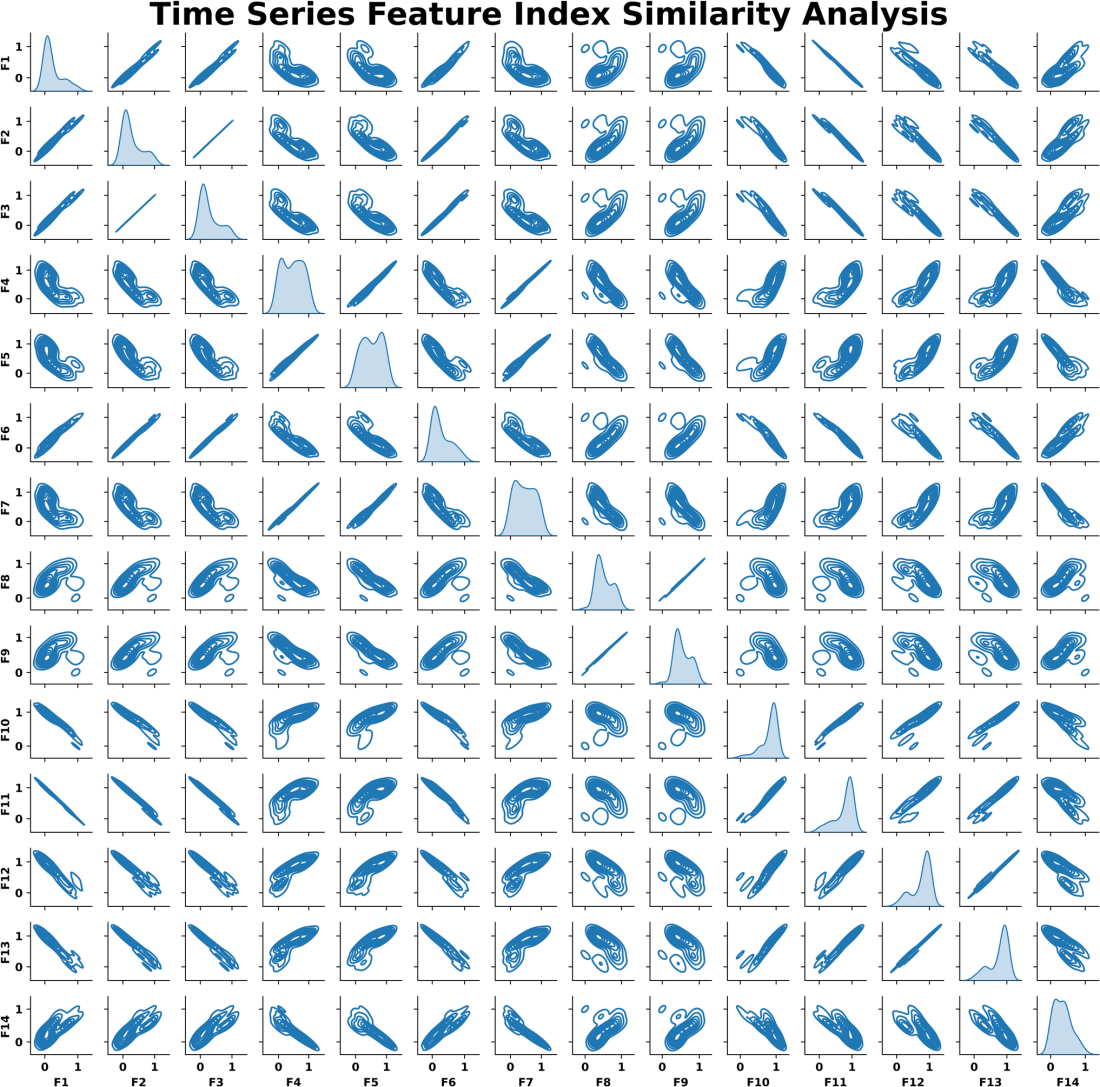
The representation ability of indicators (Full Engagement stage of the Test Group 3, Cutting Trial 10)

In this study, pairwise Kernel Density Estimation (KDE) was employed to quantitatively evaluate the linear relationships among these numerical variables, using the pair plot function from the Seaborn Python package. As depicted in Fig. 7, the diagonal elements of the pair plot illustrate univariate KDE distributions, while the off-diagonal plots present bivariate KDE contours. This visualisation not only clarifies the distributional characteristics of each factor but also elucidates their interrelationships.

The importance of this analysis becomes particularly evident when examining the cross-comparative results of the signal indicators. The summary shown in Fig. 7 highlights pronounced linear correlations among certain factors. For instance, the Shannon entropy of filtered cutting force signals—which reflects signal non-linearity—shows a marked similarity to both the Relative Entropy and Cross Entropy, employed to measure relations between filtered cutting force signals and raw measurements. Although Dynamic Time Warping (DTW) uses Euclidean distances to assess signal similarity, its computed values also demonstrate robust linear correlations with these three entropy-based factors.

Furthermore, the self-correlation of the filtered cutting force signal displays a high degree of linearity with the cross-correlation between filtered and unfiltered cutting force signals. This finding can be attributed to three main factors: (i) the preservation of dominant signal features during the filtering process, (ii) the periodic nature of cutting force signals, which reinforces strong correlations, and (iii) the mathematical proportionality between self-correlation and cross-correlation when the filtered signal closely resembles the original raw data.

Additionally, the analysis reveals a noteworthy linear relationship between Skewness and Kurtosis in the filtered cutting force signals. Although the correlation between these two factors is somewhat less pronounced than that observed between Relative Entropy and Cross Entropy, it remains comparatively strong when measured against the other factors investigated.

Despite the theoretical representations of these 14 signal features being classifiable into 10 distinct categories based on distributional similarity, all 14 factors have been retained. This choice ensures a comprehensive inclusion of feature dimensions, albeit at the cost of increased redundancy. Nevertheless, such redundancy ultimately contributes to a more robust characterisation of the target signals within the measurement system.

#### Signal complexity analysis

**Fig. 8:**
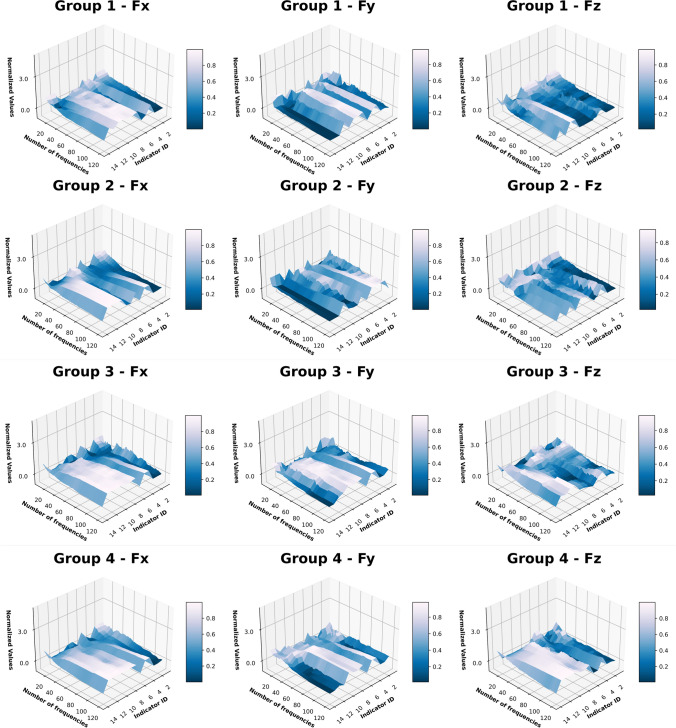
Signal indicator normalised value overview for Full Engagement stage

**Fig. 9:**
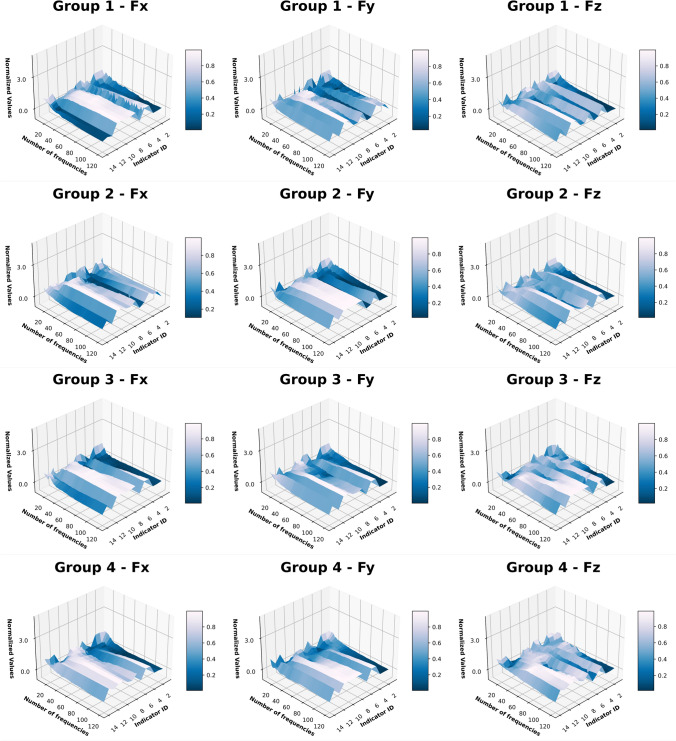
Signal indicator normalised value overview for the “Partial Engagement” stage

In Sect. “Comparison between raw signal and filtered signal”, the differences of the harmonics significancy from the FFT analysis of the “Partial Engagement” and the “Full Engagement” stages can be clearly observed. Therefore, this work will consider the number of active frequencies remained in filtering processes as well as the influences of the cutter-workpiece-engagement. The computation results of 14 factors, which have been normalised between 0 and 1, are compared with each other, as shown in Fig. 8 (“Full Engagement” stage) and Fig. 9 (“Partial Engagement” stage). To ensure the comparability crossing tests in different machining conditions, there are 20,000 force samples of each filtered signals were taken to calculate the selected indexes.

Firstly, in Figs. 8 and 9, the monotonicity can be observed in most selected factors, even if different machining parameters were applied. Among all, the Shannon entropy, relative entropy, cross entropy, and Dynamic Time Wrapping are rising with the number of band-pass frequencies. By contrast, the smoothness, Hurst exponents, volatility index and Root-Mean-Square (RMS) deviation are decreasing with the number of band-pass frequencies. Self-correlation and cross-correlation rise between 10 and 44 and climbing down afterwards with the increasing number of frequencies. From these observations, it can be concluded that the change of band-passed frequency number can affects most factors. In other words, the observed monotonicity can help with quantifying signal complexity in multiple layers.

Because of unknown interfering factors, the differences between engagement stages, axis and groups with different machining parameters applied are also obvious. In summary, there are three different types of exceptions: reversed trends, noisy trends, and collapsing demonstration.

As the number of frequency components are added, the complexity of signals increases significantly, which can be used to quantify the complexity of signals and serve as a reference for the selection of subsequent AI models.

## Signal analysis and LSTM model development

### Long-short-term-memory (LSTM) model

The Long Short-Term Memory (LSTM) model, a specialised recurrent neural network (RNN) structure, is widely utilised in the analysis of time-series data, including tasks such as time-series forecasting and natural language processing. In the context of time-series forecasting, the LSTM model continuously processes sequential data, updating its memory cells and hidden states to learn underlying patterns and dependencies. Once trained, the model can predict future values based on the learned information. LSTM addresses the vanishing and exploding gradient problems commonly encountered by traditional RNNs when handling long data sequences, making it particularly effective in tasks like time-series forecasting. This is achieved through the introduction of memory cells and gate mechanisms that regulate the flow of information, enabling the model to retain important data over long sequences (Al-Selwi et al., 2024; Waqas, 2024).

In terms of learning patterns and dependencies, this refers to the identification and extraction of inherent regularities, trends, and relationships present within time-series data. Such patterns may include periodicity, trends, and seasonality, while dependencies reflect the interactions between data points, such as the relationships between current and preceding values. For example, in the context of analysing cutting force signals—periodic time-series data influenced by cutting parameters—LSTM can effectively capture key characteristics of periodic waves, including amplitude, period, and phase. The signal may also exhibit uncertainties due to factors such as vibration, chatter, the material properties of workpieces, and thermal effects. Owing to its ability to learn patterns and dependencies, the LSTM model is well-suited to accurately predicting such signals, despite these inherent uncertainties.

Figure 11 shows the general structure of the LSTM layer and the internal structure of each LSTM cell, which includes some key components such as Input gate, Forget gate, and Output gate. The Input gate (${i}_{t}$) control the new information intakes to cell state, or it determines which values can be updated based on current input and the previous hidden state. The Forget gate (${f}_{t}$) is designed for controlling how much past information should be discarded or retained. And for the Output gate (${o}_{t}$), it controls how much of the cell state should be passed as hidden state. More specifically, the Output gate determines the final output of the LSTM unit by filtering the updated cell state through a sigmoid gate and tanh activation. The core functions of Input gate (${i}_{t}$), Forget gate (${f}_{t}$) and Output gate (${o}_{t}$) are listed in Table 3.

**Table 3:** Core equations of LSTM model

Gate Names	Formulas	Implementation
Input gate (*i*_*t*_)	${i}_{t}=\sigma ({W}_{i}\cdot \left[{h}_{t-1}, {x}_{t}\right]+{b}_{i})$	${\widetilde{C}}_{t}=\mathrm{tanh}({W}_{C}\cdot \left[{h}_{t-1}, {x}_{t}\right]+{b}_{C})$ $C_{t} = f_{t} \odot C_{t - 1} + i_{t} \odot \tilde{C}_{t}$ $h_{t} = o_{t} \odot {\mathrm{tanh}}\left( {C_{t} } \right)$
Forget gate ($${f}_{t})$$	$${f}_{t}=\sigma ({W}_{f}\cdot \left[{h}_{t-1}, {x}_{t}\right]+{b}_{f})$$	
Output gate ($${o}_{t})$$	$${o}_{t}=\sigma ({W}_{o}\cdot \left[{h}_{t-1}, {x}_{t}\right]+{b}_{o})$$	

### Applied LSTM model structure and “feature function”

The implemented RNN-DNN model comprises four main sub-structures: an input layer, an LSTM layer, a DNN layer, and an output layer. As discussed in Sect. “Long-Short-Term-Memory (LSTM) model.”, the LSTM layer demonstrates superior performance in time-series prediction, efficiently capturing both long-term and short-term features. It is particularly well-suited for modelling the periodic characteristics of the TPF and its higher-order harmonics. The attached DNN layer, which is composed of multiple fully connected layers, is specifically designed to enhance the model’s ability to detect and learn non-identical “active frequencies” present in the higher frequency bands.

**Fig. 10:**
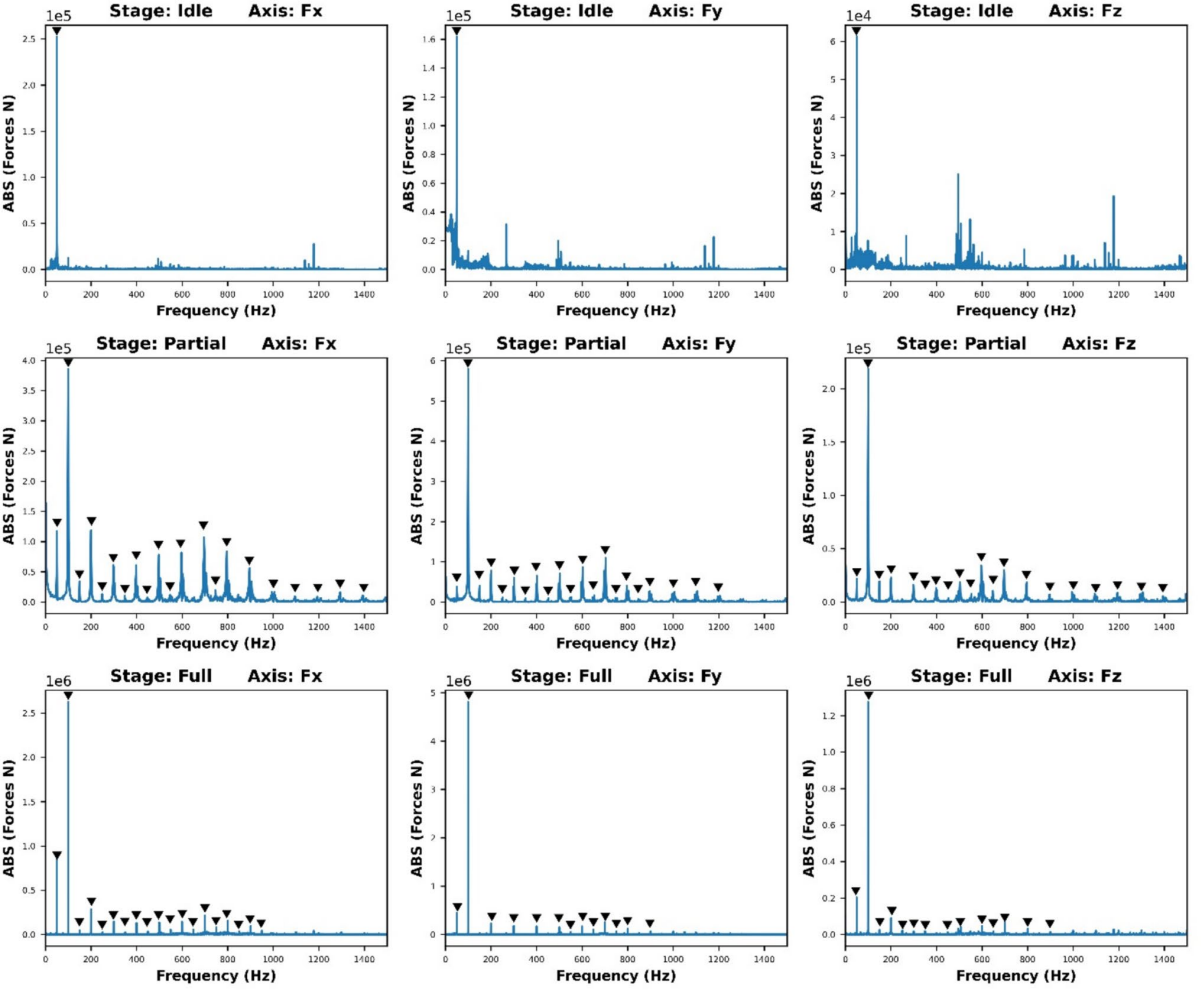
Cutting force frequency analysis at different engagement stages for Test Group 1, Trial 1

Moreover, from the signal frequency analysis of different engagement stages in Fig. 10, the carrier frequency in three axes is always TPF. Therefore, the description of TPF in training data can help reduce the training time and enhance the inference accuracy. The “feature function” based on the spindle frequency written in the form of Eq. (4) was used to generate a “feature sequence” as part of the training dataset to applied RNN-DNN models. The function aims to reduce the training time and enhance the performance of the overall prediction.

4 $$ {\mathrm{y}} = \sin \left( {2{\uppi } \cdot \left( {\frac{{\mathrm{n}}}{60}} \right) \cdot {\mathrm{x}}} \right) $$

**Fig. 11:**
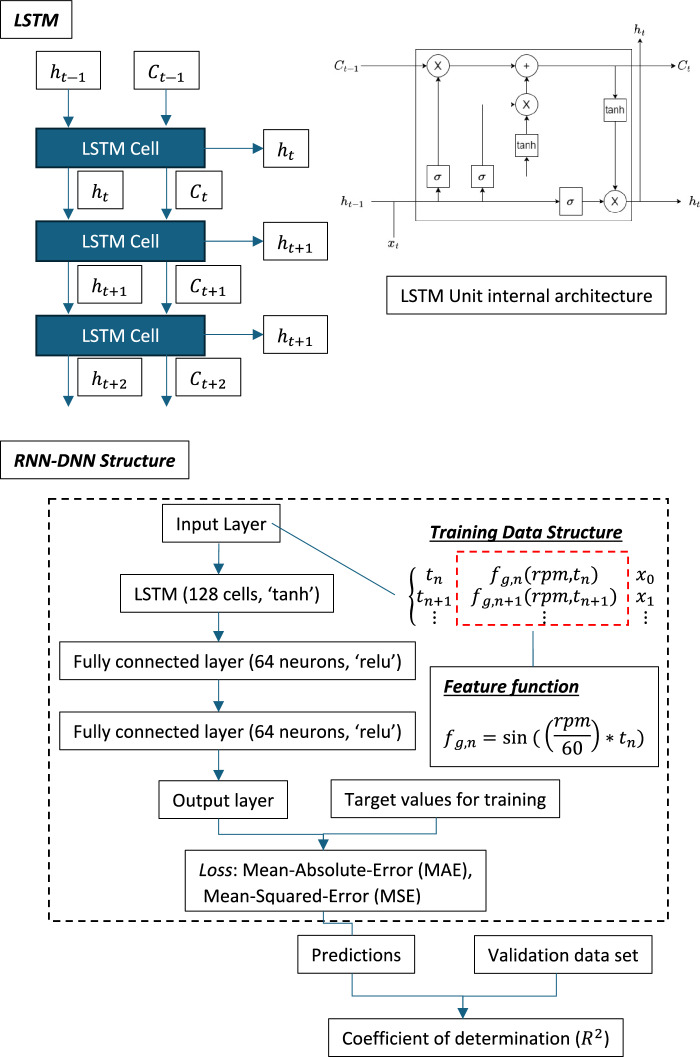
The example of LSTM model with 2 “Fully connected” layers

The structure of the RNN-DNN model is outlined in Fig. 11. Following the “Input layer,” the LSTM layer employs the tanh activation function. The tanh function is particularly effective at preventing exploding activations, capturing both positive and negative signals, and supporting long-term dependencies. All fully connected layers utilise the ReLU activation function due to its ability to prevent vanishing gradients, increase sparsity in activations, and enhance computational efficiency. Consequently, the model is structured as an LSTM-DNN architecture, with the basic configuration comprising two DNN layers. In subsequent sections, further investigations into the model’s performance will explore the use of deeper architectures, incorporating four, six, eight, and ten DNN layers.

Overall, the tooth passing frequency (TPF) derived from pre-produced G-code, is incorporated into both the training and prediction phases to enhance model performance. The TPF, a key machining parameter corresponding to the carrier frequency of the cutting force signal, guides the Long Short-Term Memory (LSTM) network to prioritise relevant frequencies. The LSTM utilises spindle speed to model the time-sequence behaviour of TPF, while Deep Neural Network (DNN) layers capture high-frequency features such as harmonics, improving signal reconstruction accuracy. By aligning the learning process with established physical principles, the model enhances interpretability while maintaining the DNN’s ability to identify complex patterns in high-frequency domains. The following study shows that this hybrid approach significantly accelerates training convergence and helps to achieve high prediction accuracy.

## General prediction performance analysis

This study explores how variations in DL model complexity affect prediction performance. The underlying hypothesis is that incorporating an increasing number of frequency components may reveal the boundaries of each model’s predictive capability. In other words, DL models with higher complexity are anticipated to capture a more comprehensive range of features. To test this hypothesis, the current work employs models constructed with a foundational architecture comprising one LSTM layer followed by varying numbers of fully connected layers. This design aims to systematically assess how changes in model complexity influence the accurate prediction of cutting forces across the active frequency spectrum, thereby informing future model design and selection.

### Average prediction performance analysis of different models

**Fig. 12:**
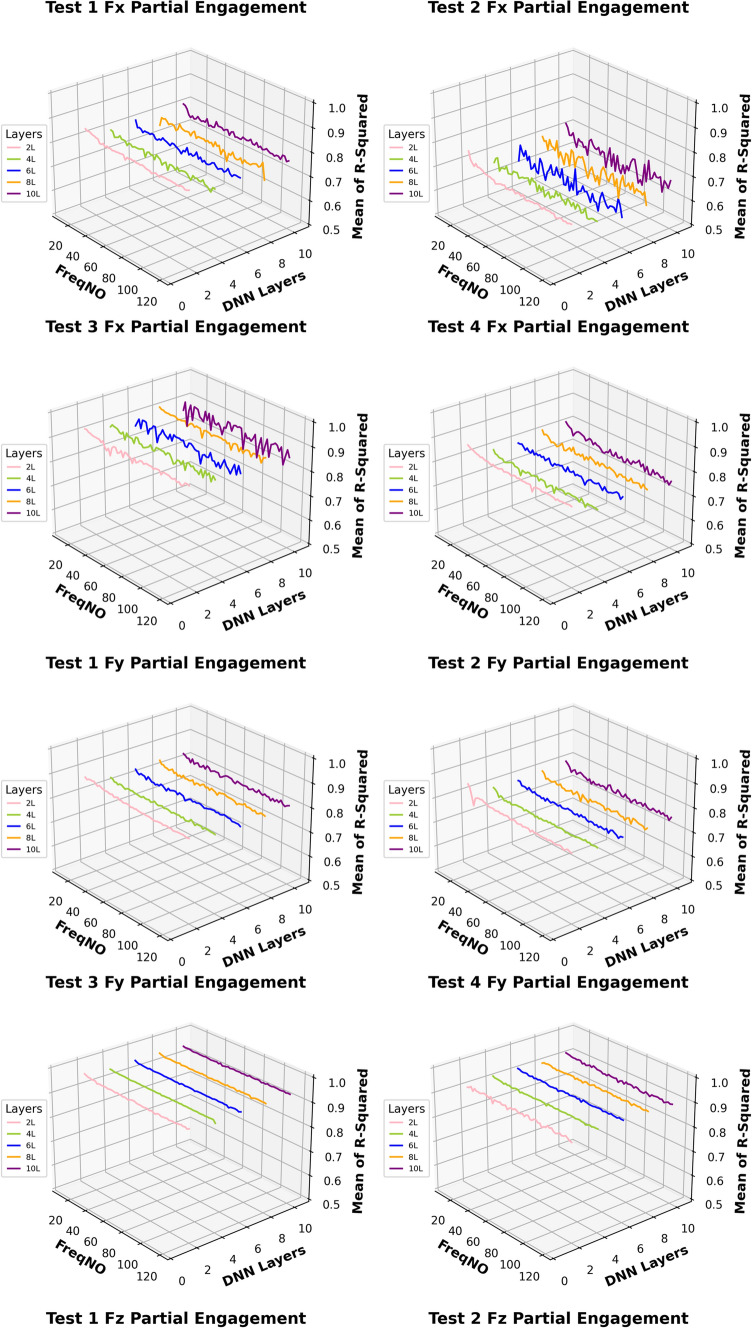

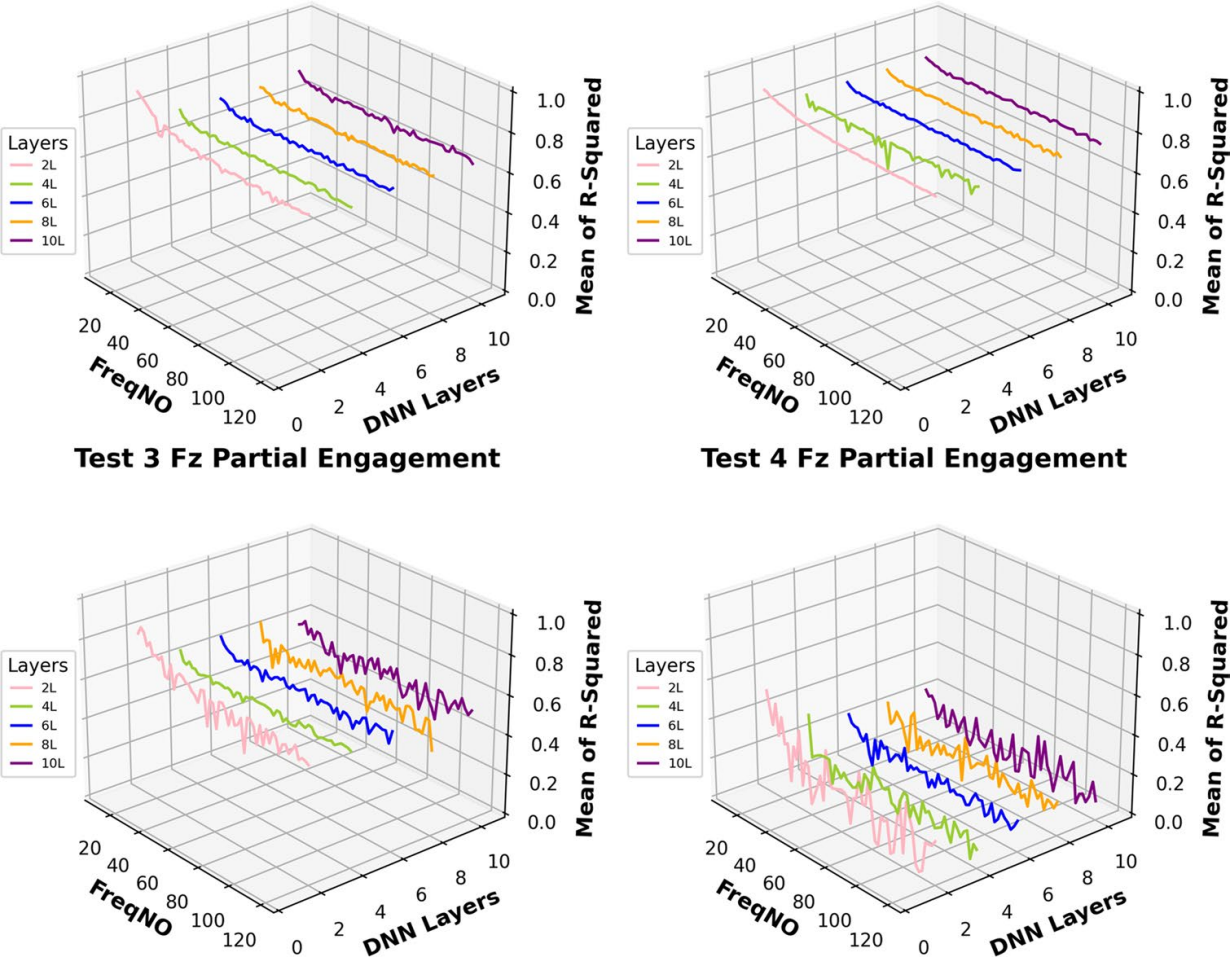
Mean of R^2^ by different layers of DNN model

This work aimed to investigate the prediction performance while applying different numbers of “fully connected” layers based on the architecture presented in Fig. 11. Figure 12 shows the prediction accuracy fluctuates of each tested model as more frequency components are added.

In general comparisons of the predictions at the “Partial Engagement” stage, the prediction curves for ${F}_{x}$ and ${F}_{y}$ are relatively more stable than those for ${F}_{z}$. The fluctuations in most ${F}_{z}$ predictions, within the observed frequency range, fall between 0.4 and 0.9. The accuracy of ${F}_{z}$ predictions for **Test Group 4** showed significantly poor performance across all tested model structures. In contrast, the differences in ${F}_{x}$ and ${F}_{y}$ predictions under the same machining conditions are less pronounced. Interestingly, the ${F}_{z}$ prediction curves for **Test Groups 1**–**3** exhibit clear trends with the increasing number of “active frequencies,” with the variations becoming gentler beyond an “active frequency” number of 60. In summary, changes to the model architecture have very limited impact on the predictions of ${F}_{x}$ and ${F}_{y}$ across all scenarios examined in this study, as well as on the number of observed “active frequencies.” In other words, the predictions for ${F}_{x}$ and ${F}_{y}$ demonstrate greater stability compared to those for ${F}_{z}$. Unlike ${F}_{x}$ and ${F}_{y}$, the models used for predicting ${F}_{z}$, which incorporate two “fully connected" layers, reveal clear dependencies on the increasing number of “active frequencies”.

**Fig. 13:**
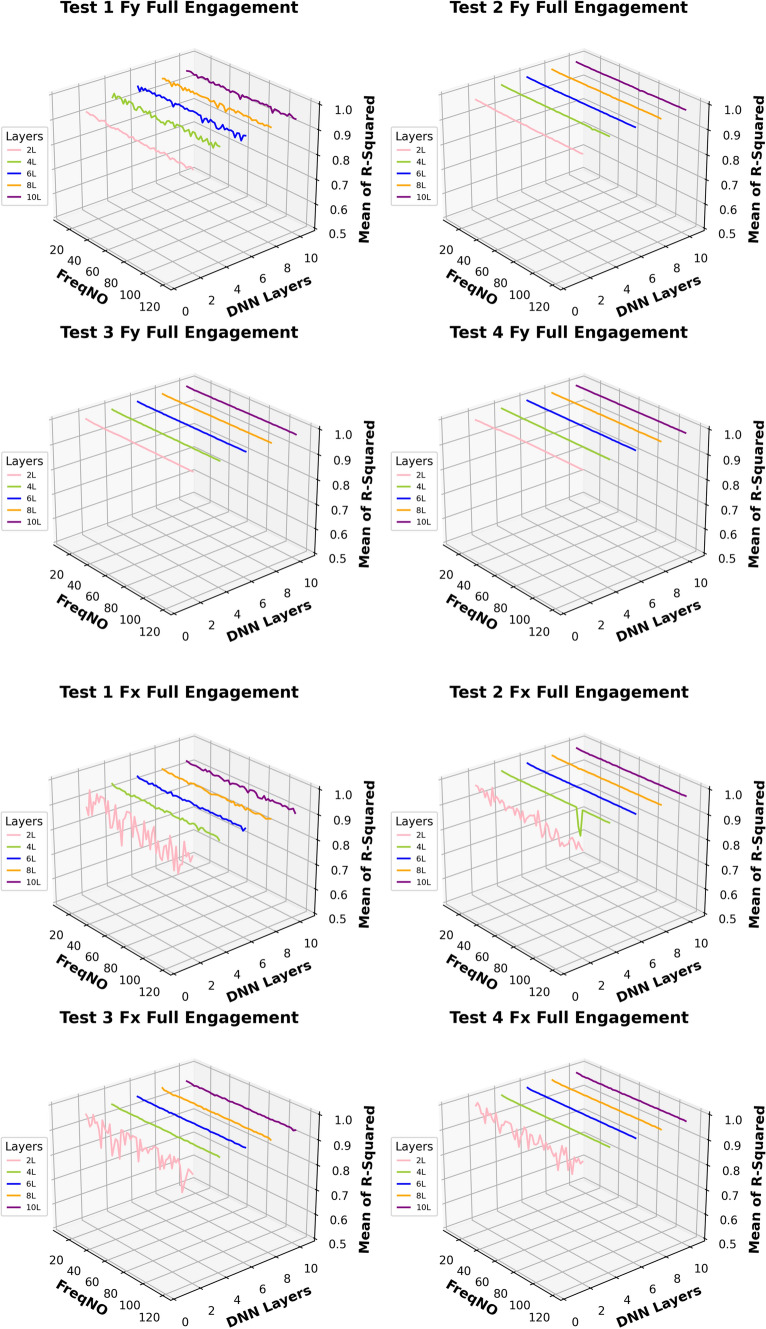

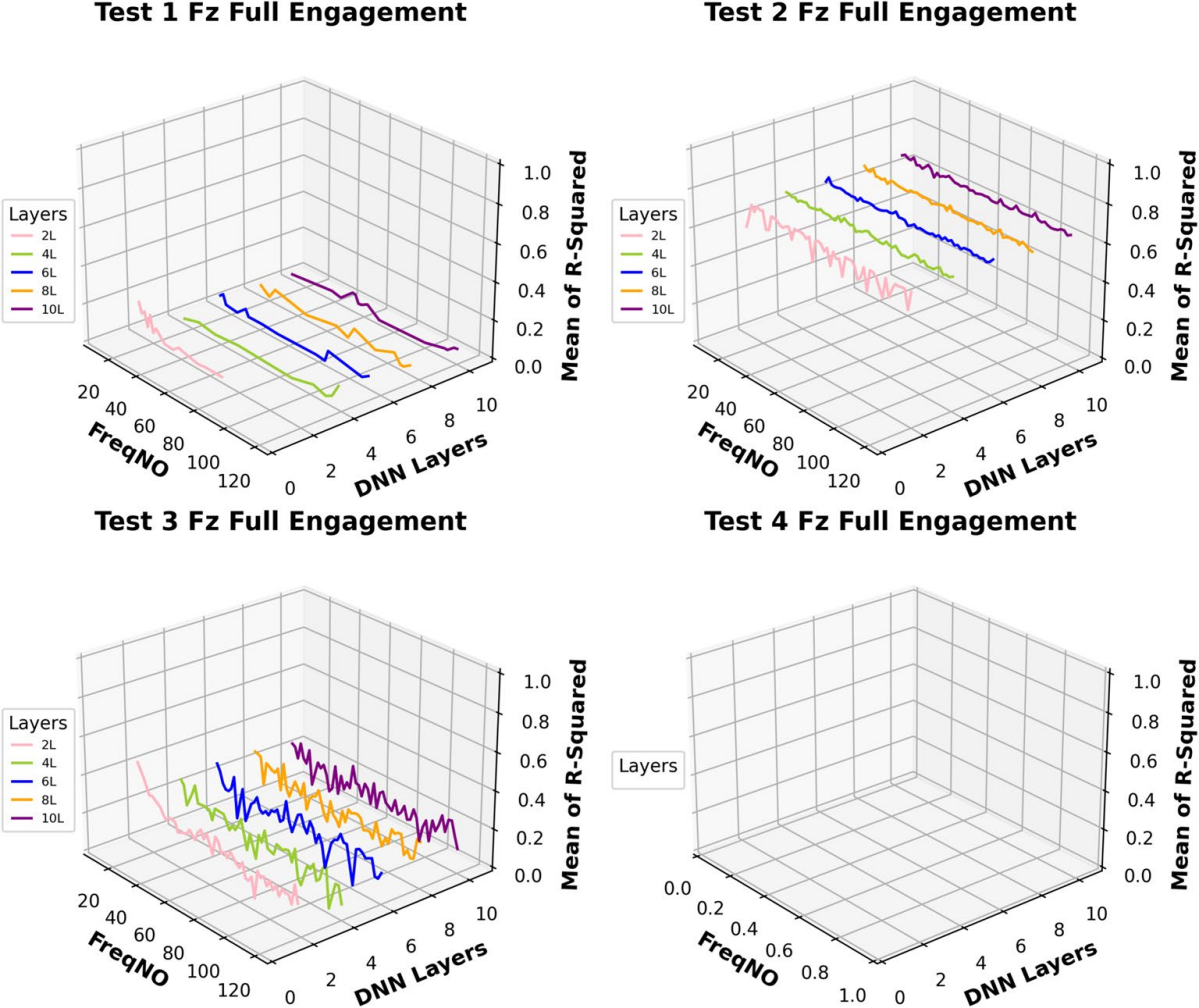
Mean of ${R}^{2}$ by different layers of DNN model

In Fig. 13, the prediction comparisons for the “Full Engagement” stage reveal a lack of dependency between the number of “active frequencies” and the number of “fully connected” layers. For the model with two “fully connected” layers, the predictions for ${F}_{x}$ vary around 0.1 as the number of observed “active frequencies” changes. A similar pattern is observed in the calculations for **Test Group 2**’s ${F}_{z}$ predictions. Surprisingly, **Test Groups 1**,** 3**, and** 4** exhibit negative ${R}^{2}$ scores, indicating a failure in predictions. As discussed in Sect. “Applied LSTM model structure and “feature function””, signal analysis shows that the amplitude and number of TPF and its higher-order harmonics are significantly lower in the “Full Engagement” stage compared to the “Partial Engagement” stage. This suggests that the proportion of “noise” in the measured ${F}_{z}$ is larger during the “Full Engagement” stage. In this task’s end milling process, the primary chip load is distributed along the X and Y axes, with minimal force in the Z direction. Consequently, in both the “Partial Engagement” and the “Full Engagement” stages, ${F}_{z}$ is significantly smaller than ${F}_{x}$ and ${F}_{y}$. However, during the “Partial Engagement” stage, the CWE varies with each cycle, which adds extra load to ${F}_{z}$. This additional force can be distinguished from “noise” by the deep learning model, explaining why most ${F}_{z}$ predictions are more accurate in the “Partial Engagement” stage than in the “Full Engagement” stage.

### Uncertainty variation of different models.

**Fig. 14:**
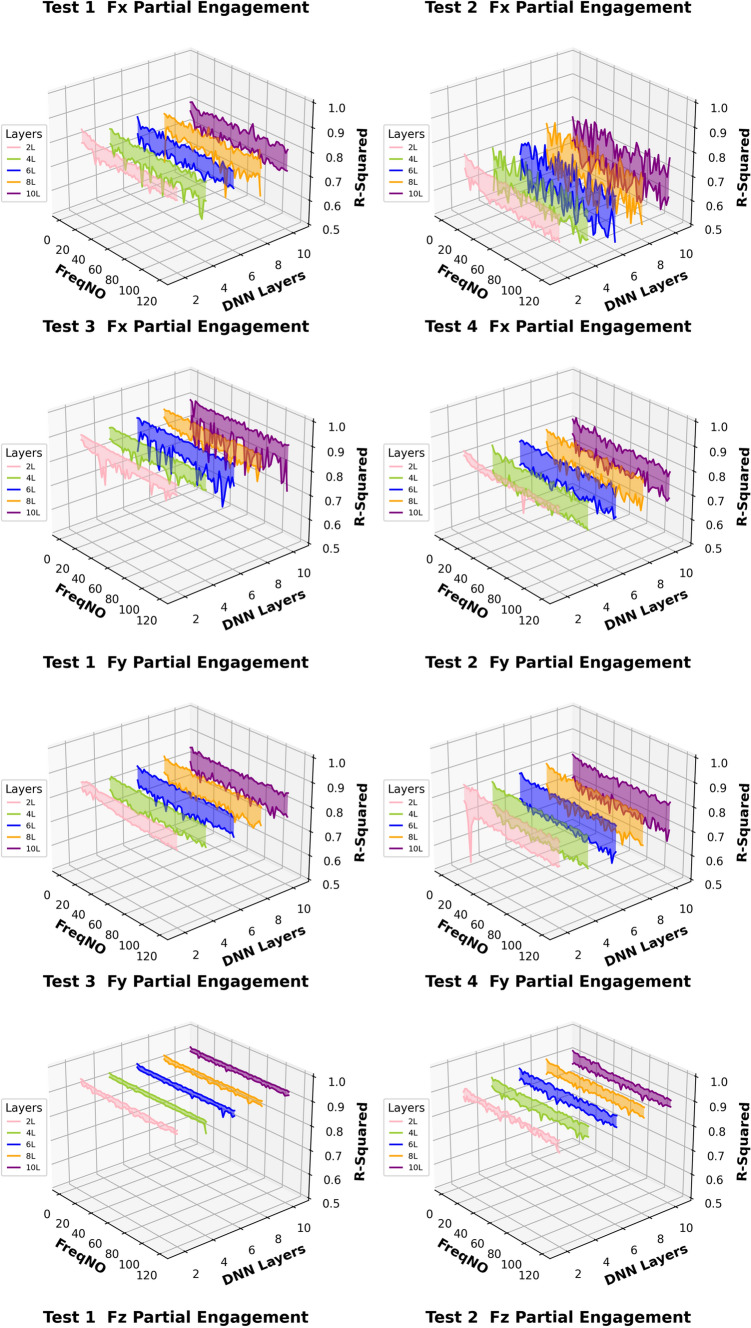

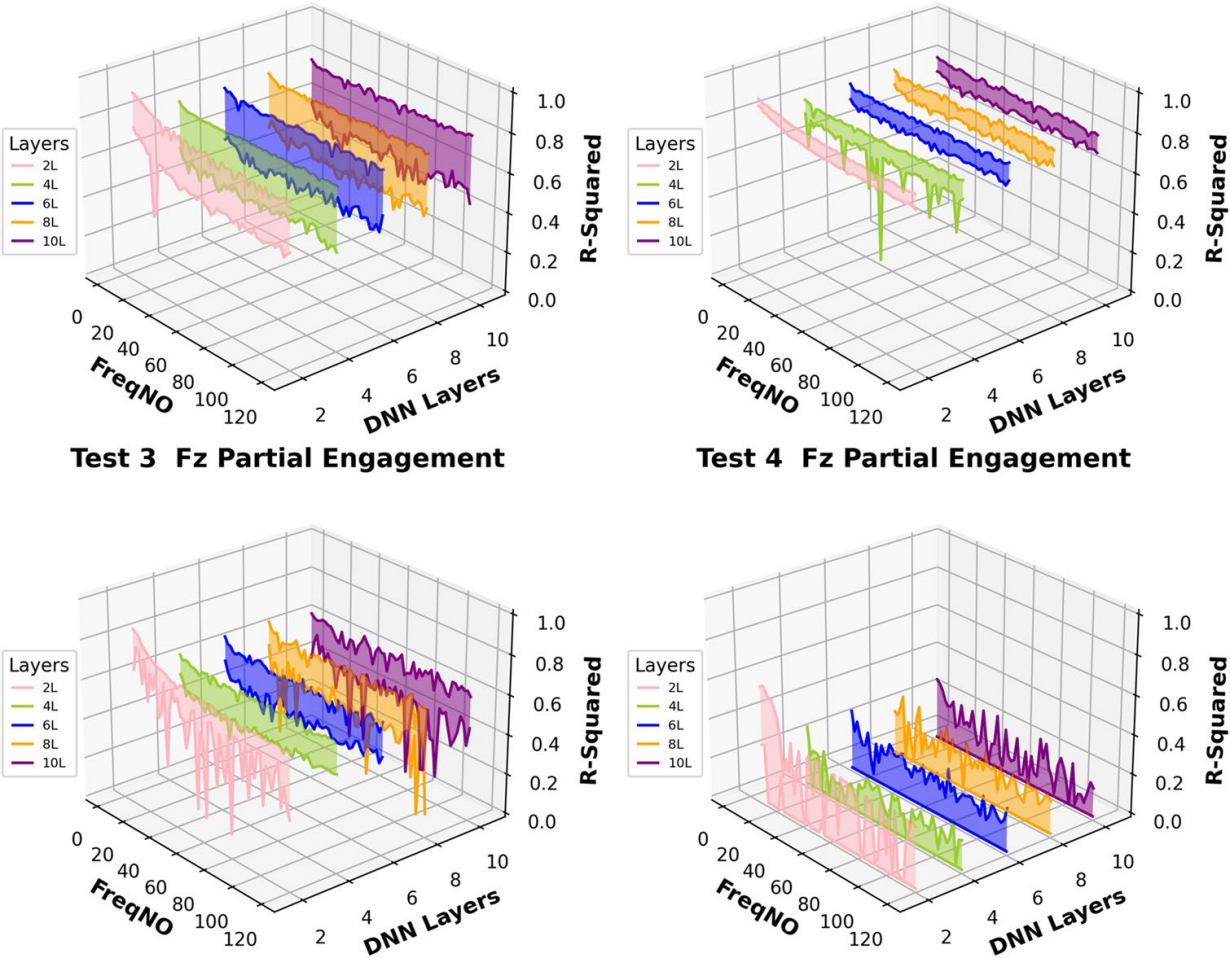
${R}^{2}$ of different models in the “Partial Engagement” stage

**Fig. 15:**
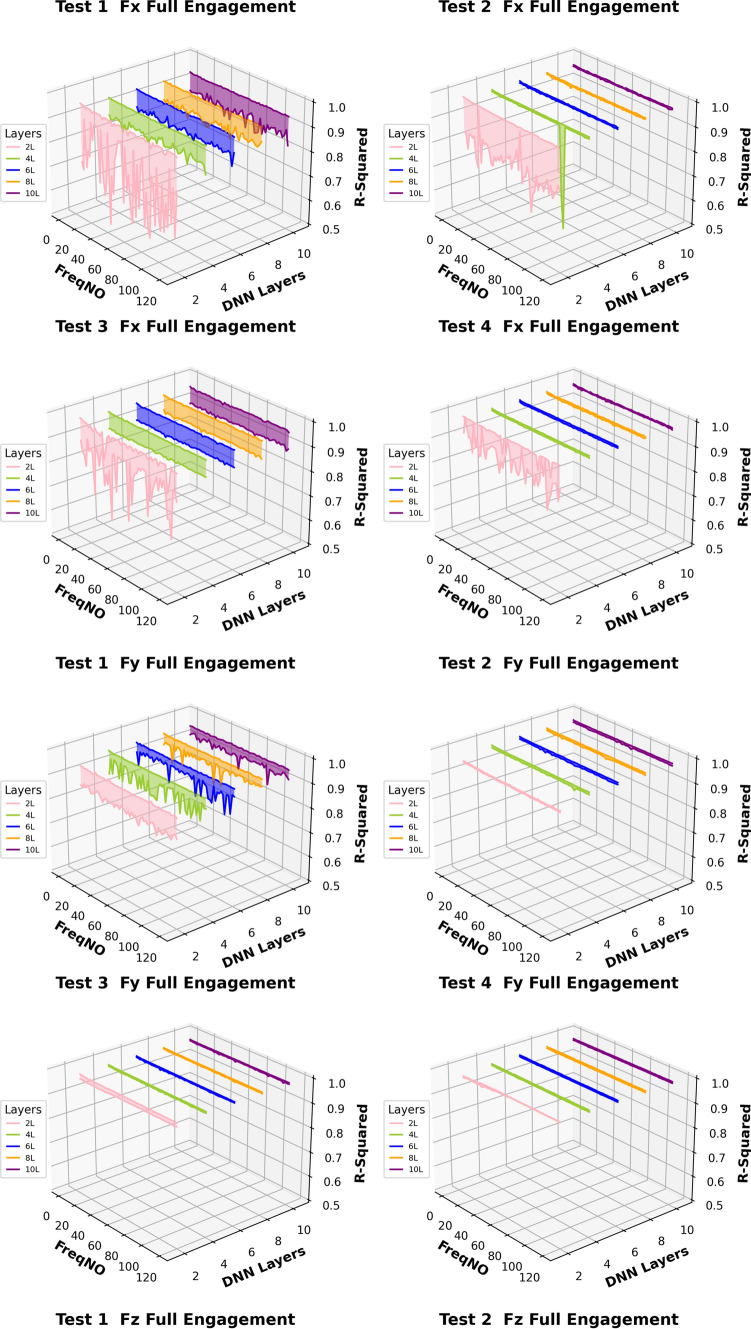

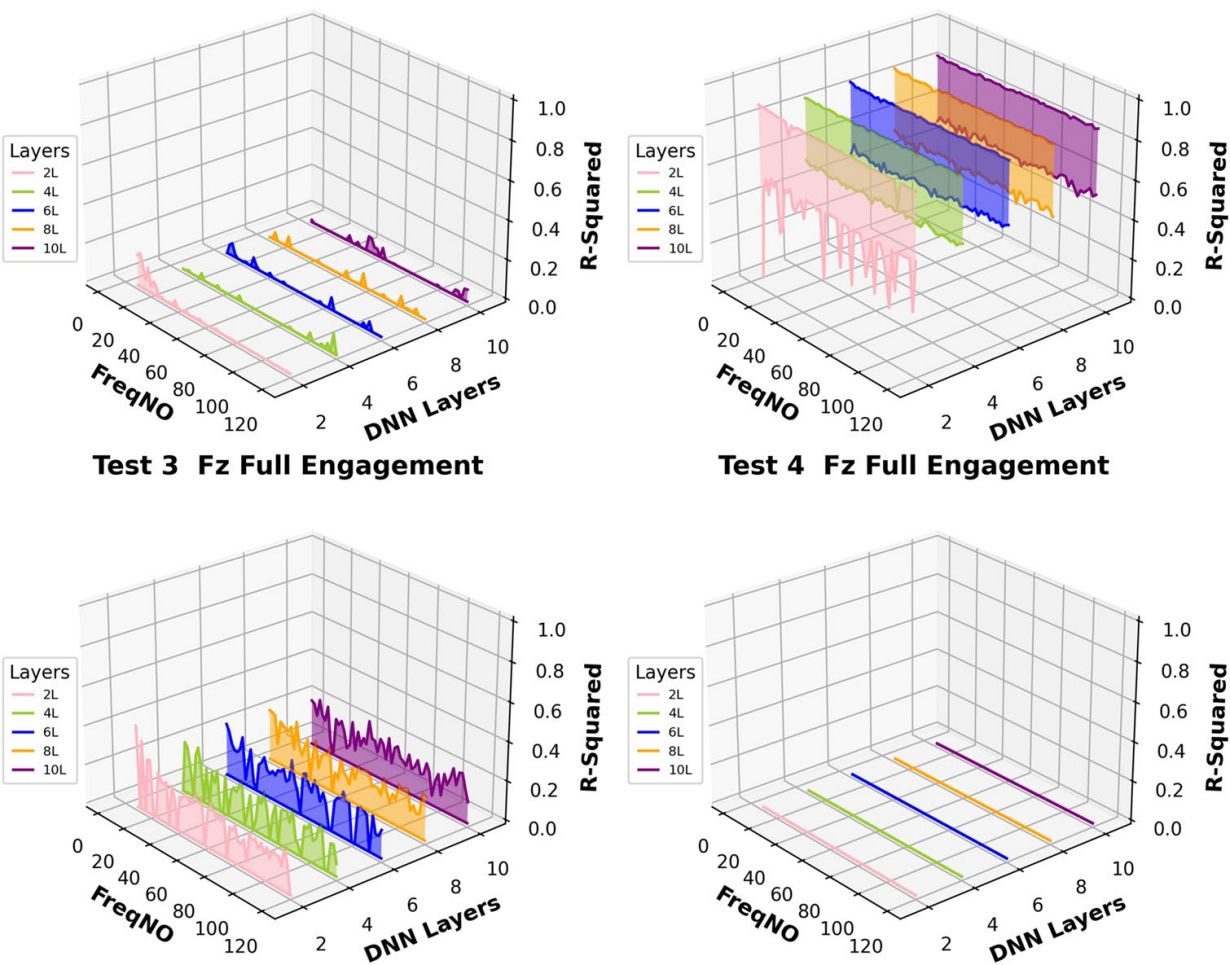
${R}^{2}$ of different models in the “Full Engagement” stage

Figures 14 and 15 present the computation of prediction uncertainty for the “Partial Engagement” and “Full Engagement” stages, with the goal of investigating the dependency of prediction uncertainty on the number of “active frequencies”.

In Fig. 14, the wide uncertainty zones are observed from most plots, which reflects the fact that the uncertainty of prediction performance variation did not rely on the changes of model architecture.

By comparing prediction uncertainty across ${F}_{x}$, ${F}_{y}$ and ${F}_{z}$, the thinnest uncertainty zones observed in the ${F}_{y}$ predictions indicate the more stable prediction performance than the predictions of the ${F}_{x}$ and the ${F}_{z}$. By taking the average variation of the ${F}_{y}$ predictions, the maximum variation can be found at the computations of **Test Group 2**, which is around 0.17, while minimum variation is found to be around 0.3 at the **Test Group 3**. From the average variation of ${F}_{z}$, regardless of poor predictions at **Test Group 4**, the prediction accuracy of the ${F}_{z}$ is the lowest, and the stability of prediction is also poorer than ${F}_{x}$ and ${F}_{y}$.

For the analysis of uncertainty variation with number of observed “active frequency”, the decreasing trends with the increase of observed “active frequency” number are observed only in the ${F}_{z}$ predictions of the **Test Groups 1**, **2** and **3**. The models with two “fully connected” layers produced sharpest descends by comparing with the models with more “fully connected” layers. And the trends stop between the number of 40–50.

The similar conclusion can be summarised from comparisons of Fig. 15, which is showing the results of the “Full Engagement” stage. The predictions of the ${F}_{y}$ is the most stable, followed by the predictions of ${F}_{x}$. The poor predictions and large uncertainty variation are observed from the predictions of the ${F}_{z}$. Unlike the results of the “Partial Engagement” stage, there is no clear trending features observed in Fig. 15.

In conclusion, this work reveals that the change of model structure has very limited influence on the uncertainty variation when the number of “fully connected” layer is set above 2. Some ${F}_{z}$ predictions at the “Partial Engagement” stage presented dependencies to the number of “active frequency” contained inside target signals, which were barely observed at the predictions of the “Full Engagement” stage. Without considering the uncertainty variation produced by the models with two “fully connected” layers, the uncertainty bands are generally thinner for the predictions of the “Full Engagement” stage but slight wider in the prediction of the “Partial Engagement” stage. From the perspective of the machining mechanism, the incremental increase in the CWE duration during each cutting cycle at the “Partial Engagement” stage induces additional vibrations, which are regarded as extraneous “noise” or a source of dataset bias. Although these noise components exert minimal influence on the performance indicators, they introduce residual uncertainties during the DL model training process. Consequently, when more than two DNN layers are applied, these uncertainties contribute to overfitting.

### Overview ${R}^{2}$ features of models with two “fully connected” layers.

The analysis of Sect. “Uncertainty variation of different models” shows that simple structure of DL model produced larger uncertainty in the process of cutting force prediction, while model with more complex structure generated better performance. And the models than two “fully connected” layers also produced similar prediction performance. Since the models with two “fully connected” layers were more “sensitive”, it is worth to furtherly study the impacts due to various factors such as the number of “active frequency” and the variation of the applied machining parameters.

#### General comparisons of predictions performances

**Fig. 16:**
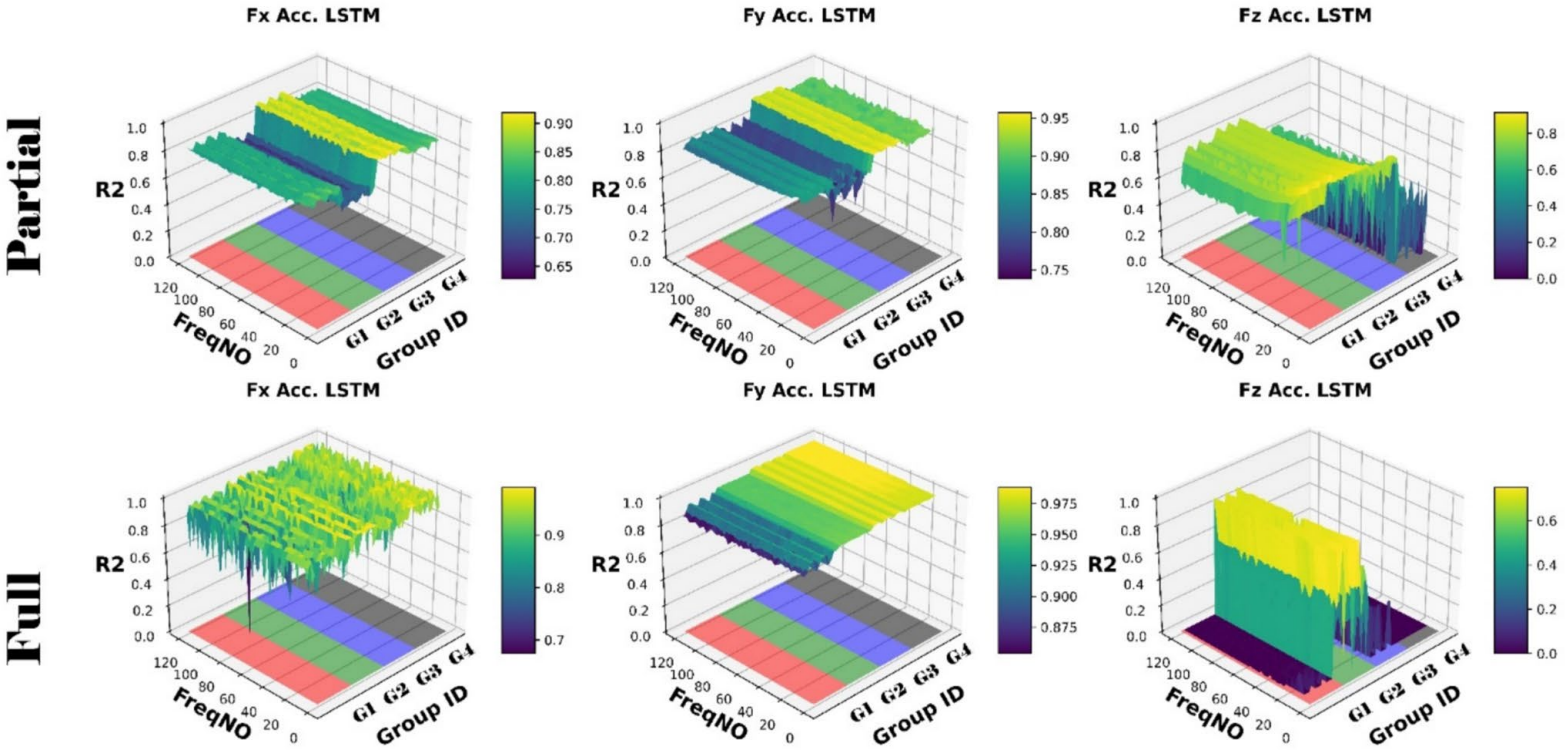
$${\mathrm{R}}^{2}$$ uncertainty analysis

Figure 16 overviews the relations of the ${R}^{2}$, the number of selected “active frequencies” and applied machining parameters. The analysis indicates ${F}_{z}$ prediction performance is significantly less than other two axes. At the “Partial Engagement” stage, the tests applied lower spindle speed (e.g., 3000 RPM) have relatively stable predictability by comparing with the tests applied higher spindle speed (e.g., 5000 RPM). The poor prediction results are observed at the most tests at the “Full Engagement” stage, except for the tests of **Test Group 2**. The ${F}_{x}$ prediction of the “Full Engagement” stage is unstable and “noisy”, unlike the ${F}_{y}$ prediction of the “Full Engagement” stage and the predictions of ${F}_{x}$ and ${F}_{y}$ at the “Partial Engagement” stage. In general, the primary loads in the cutting process are applied along the X and Y axes due to the small helix angle. Conversely, the proportion of cutting force relative to noise is higher along the Z axis, as evidenced by the frequency analysis in Fig. 10. If the TPF and its higher-order harmonics are considered key features of the cutting force, ${F}_{z}$ contains less relevant “information” regarding these features. Consequently, the lower prediction performance of ${F}_{z}$ is expected. Surprisingly, however, in comparison with the “Full Engagement” stage, the additional vibrations induced by variations in CWE during the “Partial Engagement” stage enhanced the TPF and its higher-order harmonics, thereby improving their predictability.

Moreover, the influence of machining parameters is obvious from the comparisons. For example, the maximum difference of ${R}^{2}$ between **Test Group 2** and **Test Group 3** is up to around 0.3. Because all ${F}_{y}$ predictions at the “Full Engagement” stage are very close to 1.0, the less difference of ${R}^{2}$ score can be observed.

#### Fluctuations of prediction performance for all cutting tests

**Fig. 17:**
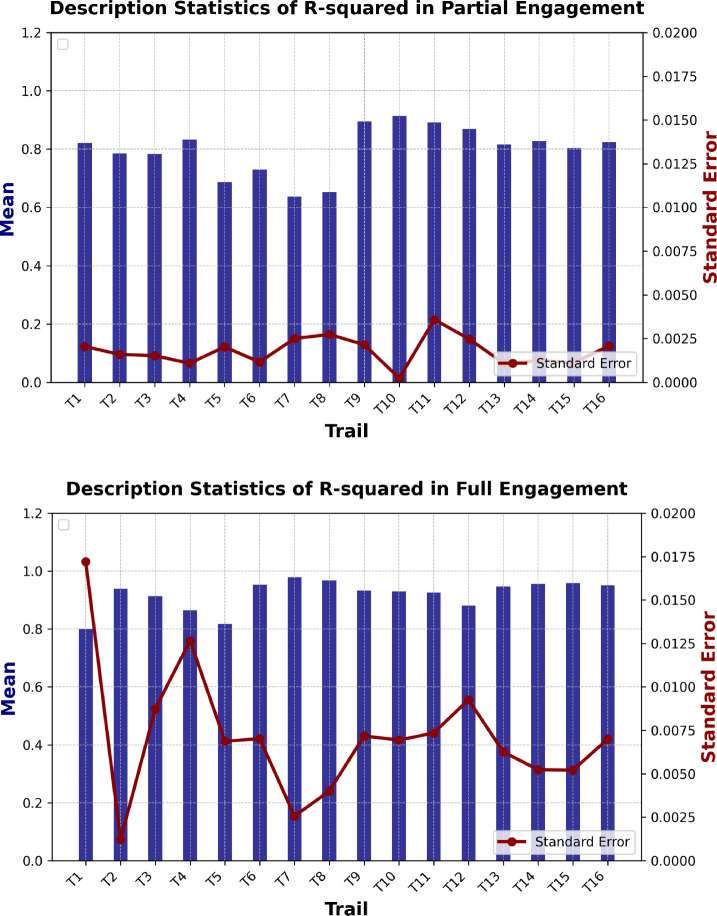
The statistics of ${R}^{2}$

To study the influence of reproducibility of machine tool system, the statistics of cutting force prediction ${R}^{2}$ score for all cutting tests are listed in Table 4 (“Partial Engagement”) and Table 5 (“Full Engagement”), and the corresponding graphs are shown in Fig. 17.

**Table 4:** ${R}^{2}$ descriptive statistics in the “Partial engagement” stage

	Test1				Test2				Test3				Test4			
*Fx in partial engagement*																
Mean	0.8211	0.7855	0.7832	0.8323	0.6876	0.7295	0.6365	0.6528	0.8952	0.9147	0.8921	0.8686	0.8169	0.8276	0.8040	0.8239
Standard error	0.0020	0.0016	0.0015	0.0011	0.0020	0.0012	0.0025	0.0027	0.0021	0.0002	0.0036	0.0025	0.0011	0.0012	0.0011	0.0021
Median	0.8199	0.7830	0.7822	0.8309	0.6869	0.7282	0.6340	0.6488	0.8997	0.9143	0.9033	0.8723	0.8149	0.8258	0.8018	0.8232
Standard deviation	0.0153	0.0119	0.0113	0.0081	0.0151	0.0086	0.0187	0.0204	0.0160	0.0017	0.0268	0.0185	0.0079	0.0093	0.0084	0.0156
Sample variance	0.0002	0.0001	0.0001	0.0001	0.0002	0.0001	0.0003	0.0004	0.0003	0.0000	0.0007	0.0003	0.0001	0.0001	0.0001	0.0002
Kurtosis	3.4727	0.9524	2.5073	1.2183	9.2620	3.3405	2.1709	5.2493	4.3071	-0.0298	3.4780	19.7943	3.6730	3.2287	4.8424	26.9141
Skewness	0.2620	0.7610	-0.1394	1.1507	2.1302	1.4722	1.1896	1.6511	-1.8650	0.7117	-1.8964	-3.8752	1.6690	0.3370	1.8608	-4.0006
Minimum	0.7678	0.7567	0.7485	0.8207	0.6637	0.7149	0.6067	0.6197	0.8330	0.9115	0.7876	0.7634	0.8049	0.7957	0.7930	0.7278
Maximum	0.8639	0.8204	0.8136	0.8558	0.7611	0.7608	0.7025	0.7413	0.9197	0.9189	0.9191	0.8985	0.8482	0.8524	0.8347	0.8537
Confidence level (95.0%)	0.0041	0.0032	0.0030	0.0022	0.0040	0.0023	0.0050	0.0055	0.0043	0.0005	0.0072	0.0049	0.0021	0.0025	0.0022	0.0042
*Fy in partial engagement*																
Mean	0.8212	0.8411	0.8357	0.8699	0.7385	0.8423	0.7943	0.7623	0.9397	0.9486	0.9472	0.9334	0.9069	0.9006	0.9024	0.9002
Standard error	0.0025	0.0016	0.0013	0.0008	0.0039	0.0014	0.0019	0.0024	0.0008	0.0007	0.0006	0.0008	0.0016	0.0012	0.0011	0.0019
Median	0.8139	0.8376	0.8375	0.8688	0.7395	0.8416	0.7905	0.7577	0.9402	0.9497	0.9469	0.9332	0.9110	0.9048	0.9024	0.9045
Standard deviation	0.0191	0.0119	0.0101	0.0059	0.0290	0.0105	0.0139	0.0177	0.0062	0.0052	0.0044	0.0063	0.0118	0.0092	0.0084	0.0140
Sample variance	0.0004	0.0001	0.0001	0.0000	0.0008	0.0001	0.0002	0.0003	0.0000	0.0000	0.0000	0.0000	0.0001	0.0001	0.0001	0.0002
Kurtosis	0.6973	− 0.2150	0.0600	1.3153	33.4601	0.0774	7.1918	8.1356	1.0699	1.1729	− 0.2933	− 0.0583	1.0534	− 0.1466	− 0.0903	− 0.2962
Skewness	1.1721	0.7989	− 0.0070	1.2255	− 4.9389	0.8328	2.3390	2.4469	− 0.3753	− 0.0724	0.1562	0.0132	− 0.8926	− 0.9132	− 0.2595	− 0.6061
Minimum	0.7968	0.8242	0.8110	0.8617	0.5503	0.8254	0.7752	0.7300	0.9232	0.9345	0.9363	0.9209	0.8688	0.8771	0.8811	0.8642
Maximum	0.8770	0.8722	0.8595	0.8869	0.7957	0.8680	0.8559	0.8365	0.9549	0.9625	0.9567	0.9502	0.9290	0.9143	0.9190	0.9223
Confidence level (95.0%)	0.0051	0.0032	0.0027	0.0016	0.0078	0.0028	0.0037	0.0047	0.0017	0.0014	0.0012	0.0017	0.0031	0.0025	0.0022	0.0038
*Fz in partial engagement*																
Mean	0.5872	0.7342	0.7891	0.8188	0.8056	0.8540	0.7926	0.8056	0.6797	0.6627	0.6505	0.5470	− 0.4184	− 0.5246	− 0.1039	0.1218
Standard error	0.0093	0.0069	0.0048	0.0070	0.0036	0.0030	0.0042	0.0035	0.0089	0.0100	0.0137	0.0207	0.1038	0.0878	0.0567	0.0482
Median	0.5761	0.7128	0.7740	0.8082	0.7951	0.8480	0.7796	0.7962	0.6443	0.6670	0.6741	0.5825	− 0.2435	− 0.3751	0.0299	0.3001
Standard deviation	0.0694	0.0514	0.0362	0.0527	0.0270	0.0222	0.0313	0.0263	0.0663	0.0748	0.1022	0.1548	0.7771	0.6573	0.4245	0.3575
Sample variance	0.0048	0.0026	0.0013	0.0028	0.0007	0.0005	0.0010	0.0007	0.0044	0.0056	0.0104	0.0240	0.6039	0.4320	0.1802	0.1278
Kurtosis	2.5707	3.5916	2.1847	0.3944	0.5890	0.4520	0.9357	2.9683	1.3906	2.9432	3.9437	1.3778	2.5248	2.8692	2.7634	0.4679
Skewness	0.1582	1.9486	1.6726	0.4100	1.2282	1.1565	1.4313	1.7665	1.5148	− 0.7205	− 1.7278	− 1.1165	− 1.4066	− 1.5442	− 1.7679	− 1.1486
Minimum	0.3722	0.6672	0.7540	0.6888	0.7777	0.8297	0.7620	0.7781	0.6236	0.4011	0.2672	0.1044	− 3.2707	− 2.7770	− 1.6128	− 0.8554
Maximum	0.7721	0.9127	0.9025	0.9427	0.8808	0.9138	0.8762	0.8936	0.8717	0.8298	0.8174	0.7862	0.5956	0.5614	0.4315	0.5838
Confidence level (95.0%)	0.0186	0.0138	0.0097	0.0141	0.0072	0.0059	0.0084	0.0070	0.0178	0.0200	0.0274	0.0415	0.2081	0.1760	0.1137	0.0966

**Table 5:** ${R}^{2}$ Descriptive statistics in “Full Engagement” stage

	Test1				Test2				Test3				Test4			
*Fx in full engagement*																
Mean	0.8005	0.9386	0.9136	0.8651	0.8175	0.9526	0.9782	0.9678	0.9320	0.9294	0.9256	0.8807	0.9469	0.9557	0.9584	0.9510
Standard error	0.0172	0.0012	0.0087	0.0127	0.0069	0.0070	0.0026	0.0040	0.0072	0.0069	0.0073	0.0093	0.0063	0.0052	0.0052	0.0070
Median	0.8803	0.9358	0.9522	0.9436	0.8124	0.9825	0.9803	0.9782	0.9715	0.9593	0.9540	0.9066	0.9818	0.9857	0.9833	0.9890
Standard deviation	0.1287	0.0090	0.0654	0.0948	0.0514	0.0525	0.0192	0.0299	0.0537	0.0519	0.0550	0.0693	0.0468	0.0391	0.0389	0.0524
Sample variance	0.0166	0.0001	0.0043	0.0090	0.0026	0.0028	0.0004	0.0009	0.0029	0.0027	0.0030	0.0048	0.0022	0.0015	0.0015	0.0027
Kurtosis	− 1.3257	1.3685	− 0.3171	− 1.3393	3.1313	− 0.2129	33.1205	3.0297	− 1.1360	1.2438	2.6044	4.4769	− 0.9897	− 0.9371	− 0.2025	0.1734
Skewness	− 0.6953	1.2697	− 1.1280	− 0.5166	0.7486	− 1.2062	− 5.5884	− 2.1595	− 0.6789	− 1.4613	− 1.8019	− 2.3923	− 0.7433	− 0.7296	− 1.0756	− 1.0529
Minimum	0.5331	0.9269	0.7441	0.6822	0.6701	0.8259	0.8557	0.8814	0.8126	0.7622	0.7327	0.6350	0.8479	0.8672	0.8505	0.7936
Maximum	0.9247	0.9671	0.9653	0.9530	0.9704	0.9931	0.9909	0.9894	0.9875	0.9719	0.9651	0.9184	0.9923	0.9937	0.9906	0.9952
Confidence level (95.0%)	0.0345	0.0024	0.0175	0.0254	0.0138	0.0141	0.0051	0.0080	0.0144	0.0139	0.0147	0.0186	0.0125	0.0105	0.0104	0.0140
*Fy in full engagement*																
Mean	0.8545	0.8801	0.9360	0.9290	0.9573	0.9534	0.9558	0.9541	0.9828	0.9835	0.9854	0.9690	0.9860	0.9862	0.9849	0.9856
Standard Error	0.0016	0.0013	0.0005	0.0005	0.0003	0.0003	0.0003	0.0003	0.0002	0.0001	0.0001	0.0002	0.0004	0.0005	0.0004	0.0006
Median	0.8562	0.8817	0.9351	0.9288	0.9563	0.9529	0.9557	0.9538	0.9832	0.9833	0.9855	0.9696	0.9872	0.9883	0.9866	0.9880
Standard deviation	0.0121	0.0101	0.0041	0.0039	0.0022	0.0023	0.0026	0.0020	0.0011	0.0007	0.0008	0.0014	0.0027	0.0038	0.0028	0.0045
Sample variance	0.0001	0.0001	0.0000	0.0000	0.0000	0.0000	0.0000	0.0000	0.0000	0.0000	0.0000	0.0000	0.0000	0.0000	0.0000	0.0000
Kurtosis	0.7071	0.6620	1.1794	1.5300	0.5085	0.2827	− 1.1172	0.5738	1.7929	11.1850	3.8579	− 0.3384	4.3579	0.9895	− 1.4964	0.0732
Skewness	− 0.5773	− 0.4792	0.8987	0.9531	1.2497	0.8075	0.2232	0.9912	− 1.6796	2.9753	0.9186	− 0.9349	− 2.2012	− 1.5397	− 0.5167	− 1.3586
Minimum	0.8204	0.8537	0.9283	0.9223	0.9538	0.9491	0.9517	0.9510	0.9795	0.9823	0.9837	0.9655	0.9755	0.9749	0.9793	0.9733
Maximum	0.8776	0.9023	0.9486	0.9427	0.9628	0.9600	0.9608	0.9596	0.9839	0.9867	0.9884	0.9710	0.9879	0.9888	0.9877	0.9886
Confidence level (95.0%)	0.0032	0.0027	0.0011	0.0011	0.0006	0.0006	0.0007	0.0005	0.0003	0.0002	0.0002	0.0004	0.0007	0.0010	0.0008	0.0012
*Fz in full engagement*																
Mean	− 0.0196	− 0.5044	− 0.0472	− 0.3723	0.8892	0.5498	0.5889	0.5456	− 0.8375	− 5.3417	− 0.8503	− 0.4759	− 2.6232	− 2.1806	− 2.2432	− 1.1296
Standard error	0.0081	0.0063	0.0040	0.0061	0.0031	0.0039	0.0201	0.0093	0.3747	0.6003	0.2395	0.0433	0.1287	0.1234	0.0532	0.0762
Median	− 0.0329	− 0.5080	− 0.0485	− 0.3664	0.8899	0.5595	0.6709	0.5506	0.1736	− 6.0550	− 0.3404	− 0.3497	− 2.4070	− 1.8279	− 2.2229	− 0.9697
Standard deviation	0.0607	0.0472	0.0302	0.0456	0.0231	0.0292	0.1503	0.0697	2.8042	4.4921	1.7922	0.3243	0.9630	0.9237	0.3983	0.5703
Sample variance	0.0037	0.0022	0.0009	0.0021	0.0005	0.0008	0.0226	0.0049	7.8634	20.1787	3.2119	0.1052	0.9273	0.8532	0.1587	0.3253
Kurtosis	2.4335	− 0.3653	− 0.0341	4.1571	26.4457	1.6479	0.1857	52.2179	7.0292	0.5368	11.3760	6.7965	0.1736	2.8174	2.4435	2.3445
Skewness	1.4181	0.1821	0.0737	− 1.2570	− 4.3876	− 1.6151	− 1.3246	− 7.1012	− 2.8405	− 0.9254	− 3.4166	− 2.4813	− 0.9829	− 1.7647	− 1.2263	− 1.6552
Minimum	− 0.1078	− 0.5924	− 0.1185	− 0.5613	0.7470	0.4639	0.2141	0.0416	− 10.8529	− 19.2938	− 9.3550	− 1.7720	− 5.0788	− 5.2173	− 3.6754	− 2.8639
Maximum	0.1768	− 0.3880	0.0346	− 0.2888	0.9191	0.5822	0.7153	0.5923	0.4203	− 1.0144	− 0.0921	− 0.1136	− 1.3060	− 1.2213	− 1.5549	− 0.3827
Confidence level (95.0%)	0.0163	0.0126	0.0081	0.0122	0.0062	0.0078	0.0402	0.0187	0.7510	1.2030	0.4800	0.0869	0.2579	0.2474	0.1067	0.1527

As for Fig. 13 illustrate, the analysis of prediction performance across the cutting tests, segmented into “Partial Engagement” and “Full Engagement”—reveals significant differences in mean and standard errors. For the Partial Engagement, the mean ranges from 0.636 to 0.914, with an overall average of approximately 0.798. The standard errors are relatively low, indicating a stable performance, with the highest standard error 0.0025 observed in Trail 7, suggesting some fluctuations. In contrast, the Full Engagement exhibits higher mean values, ranging from 0.800 to 0.978, with a higher average of approximately 0.919. This suggests that the Full Engagement tests generally perform better in terms of prediction accuracy. However, the standard errors are more variable, particularly 0.0069 in Trail 5 and 0.0093 in Trail 12, indicating greater fluctuations in performance under these conditions. Overall, the Full Engagement Tests, particularly Trails 6 to 8, shows the best prediction performance, while Trails 5 and 6 in Partial Engagement exhibit the least stability, indicated by their lower mean and higher variability in performance. The results underscore the critical role of engagement conditions in machining processes, demonstrating that Full Engagement contributes to enhanced consistency and reliability in predictive outcomes. In contrast, the analysis reveals the inherent variability associated with Partial Engagement, suggesting that this condition may introduce greater uncertainty in the prediction performance.

From the statistics presented in Tables 4 and 5, it is evident that the predictions of ${F}_{z}$ are significantly affected by system uncertainty. Notably, during the “Partial Engagement” stage, the prediction variation reaches as high as 0.6464 (considering negative ${R}^{2}$), while for the "Full Engagement" stage, this variation increases to 4.8658 (also considering negative ${R}^{2}$). A similar conclusion can be drawn from the statistical measures of “Standard Error”, “Median”, “Standard Deviation”, and “Sample Variance’. In the “Full Engagement” stage, the maximum variation in ${F}_{z}$ for these four indicators exceeds that of ${F}_{x}$ by more than 30 times and is at least 60 times higher than the corresponding variation in ${F}_{y}$. During the “Partial Engagement” stage, the differences in maximum prediction variation between ${F}_{x}$, ${F}_{y}$, and ${F}_{z}$ are all below 0.2.

Regarding the kurtosis and skewness analysis in Tables 4 and 5, the “Full Engagement” analysis reveals that the ${F}_{x}$ axis shows **Test Group 1** with a kurtosis of − 1.3257, indicating a flat, left-skewed distribution that results in poor ${R}^{2}$ values due to insufficient data concentration. In contrast, **Test Group 2** exhibits a kurtosis of 1.3685 and positive skewness of 1.2697, enhancing ${R}^{2}$ values and model adaptability. **Test Group 3**, with a kurtosis of -0.3171 and negative skewness of -1.1280, displays uneven data distribution that adversely affects predictive performance. Similarly, **Test Group 4**’s kurtosis of − 1.3393 and positive skewness of − 0.5166 also limit performance. For the ${F}_{y}$ axis, **Test Group 1** has a kurtosis of 0.7071 with negative skewness (− 0.5773), while **Test Group** 2 demonstrates a kurtosis of 0.6620 and a similar negative skew, both indicating left-skewed data. **Test Group 3** offers improved distribution (kurtosis of 1.1794 and positive skewness of 0.8987), but **Test Group 4** excels with a kurtosis of 1.5300 and positive skewness, effectively capturing higher ${R}^{2}$ values. In the ${F}_{z}$ analysis, **Test Group 1** exhibits favourable characteristics (kurtosis of 2.4335 and positive skewness of 1.4181), although ${R}^{2}$ values remain low. **Test Group 2** shows a kurtosis of − 0.3653 and negative skewness, limiting its performance, while in **Test Group 4**, the kurtosis of 4.1571 indicates significant left skewness, affecting ${R}^{2}$ capture. In the Partial Engagement analysis, **Test Groups 1** and **3** for ${F}_{x}$ present negative kurtosis values, indicating flat distributions, whereas **Test Group 2** performs better with positive skewness and kurtosis. In ${F}_{y}$ **Test Group 4** significantly enhances ${R}^{2}$.

In summary, the overall statistics show that the predictions at the “Partial Engagement” stage are relatively stable compared to those in the “Full Engagement” stage. System uncertainty profoundly impacts predictions for ${F}_{z}$. For ${F}_{x}$ and ${F}_{y}$ predictions at the “Full Engagement” stage, **Test Groups 2** and **4** consistently outperform **Test Groups 1** and **3** across various axes.

#### Relations with machining parameters applied

Overall, the applied RNN-DNN models have demonstrated promising predictive performance, as shown in previous analyses. Numerous studies suggest that variations in machining parameters can impact cutting force predictions. This section investigates the influence of such parameters on cutting force prediction. However, it should be noted that as the specific case study, the following analysis and numeric features are limited to the applied materials of cutter and workpieces, and the machining task setup in this work.

Table 6 summarises the effects of cyclic feed rate and spindle speed, building on the data from Tables 4 and 5. According to the statistics, variation in feed rate does not exert a significant influence on the prediction results. In the “Partial Engagement” stage, the highest ${R}^{2}$ scores for ${F}_{x}$ (0.8927) and ${F}_{y}$ (0.9422) were observed in **Test Group 3**, where a feed rate of 0.1600 mm/rev was applied. Conversely, the lowest scores (${F}_{x}$: 0.6766, ${F}_{y}$: 0.7843) were recorded in **Test Group 2**, which utilised a feed rate of 0.25 mm/rev. Although better predictions were observed in the “Full Engagement” stage, no clear relationship between feed rate variation and the average ${R}^{2}$ values of the predictions could be established.

**Table 6:** Feed rate vs. average ${R}^{2}$ (considering negative value)

Test group	Test 1	Test 2	Test 3	Test 4	Engagement
Feed rate (mm/rev)	0.1333	0.2500	0.1600	0.3000	
${{\boldsymbol{F}}}_{{\boldsymbol{x}}}$	0.8055	0.6766	0.8927	0.8181	Partial
${{\boldsymbol{F}}}_{{\boldsymbol{y}}}$	0.8420	0.7843	0.9422	0.9025	
${{\boldsymbol{F}}}_{{\boldsymbol{z}}}$	0.7323	0.8144	0.6349	− 0.2313	
${{\boldsymbol{F}}}_{{\boldsymbol{x}}}$	0.8794	0.9290	0.9169	0.9530	Full
${{\boldsymbol{F}}}_{{\boldsymbol{y}}}$	0.8999	0.9551	0.9802	0.9857	
${{\boldsymbol{F}}}_{{\boldsymbol{z}}}$	− 0.2359	0.6434	− 1.8764	− 2.0442	
RPM	3000		5000		

In contrast to feed rate, spindle speed was shown to have a clear influence on predictions. When comparing the average ${R}^{2}$ scores of **Test Groups 3** and **4** with those of **Test Groups 1** and **2**, for most test cases, it was evident that the groups using a spindle speed of 5000 RPM achieved higher ${R}^{2}$ scores than those using 3000 RPM in both the “Partial Engagement” and “Full Engagement” stages. In the “Partial Engagement” stage, the differences in ${R}^{2}$ scores for ${F}_{x}$ and ${F}_{y}$ were 0.1143 and 0.1092, respectively. In the “Full Engagement” stage, these differences were less pronounced, at 0.0307 for ${F}_{x}$ and 0.0554 for ${F}_{y}$.

Finally, the predictive performance of ${F}_{z}$ at both engagement stages lacks confidence and stability, making it unsuitable for studying the influence of machining parameters.

For the **Test Groups 1** and **2**, the lower cutting speeds and feed rates may restrict energy release during the cutting process, resulting in lower ${R}^{2}$ values. In contrast, **Test Groups 3** and** 4** operate at RPM of 5000 with feed rates of 800 mm/m and 1500 mm/m, enhancing cutting efficiency and yielding higher ${R}^{2}$, reflective of more effective data distribution characteristics. These observations underscore the significant differences in prediction performance based on the selected machining parameters.

### Correlation between selected indicators and prediction performance

**Fig. 18:**
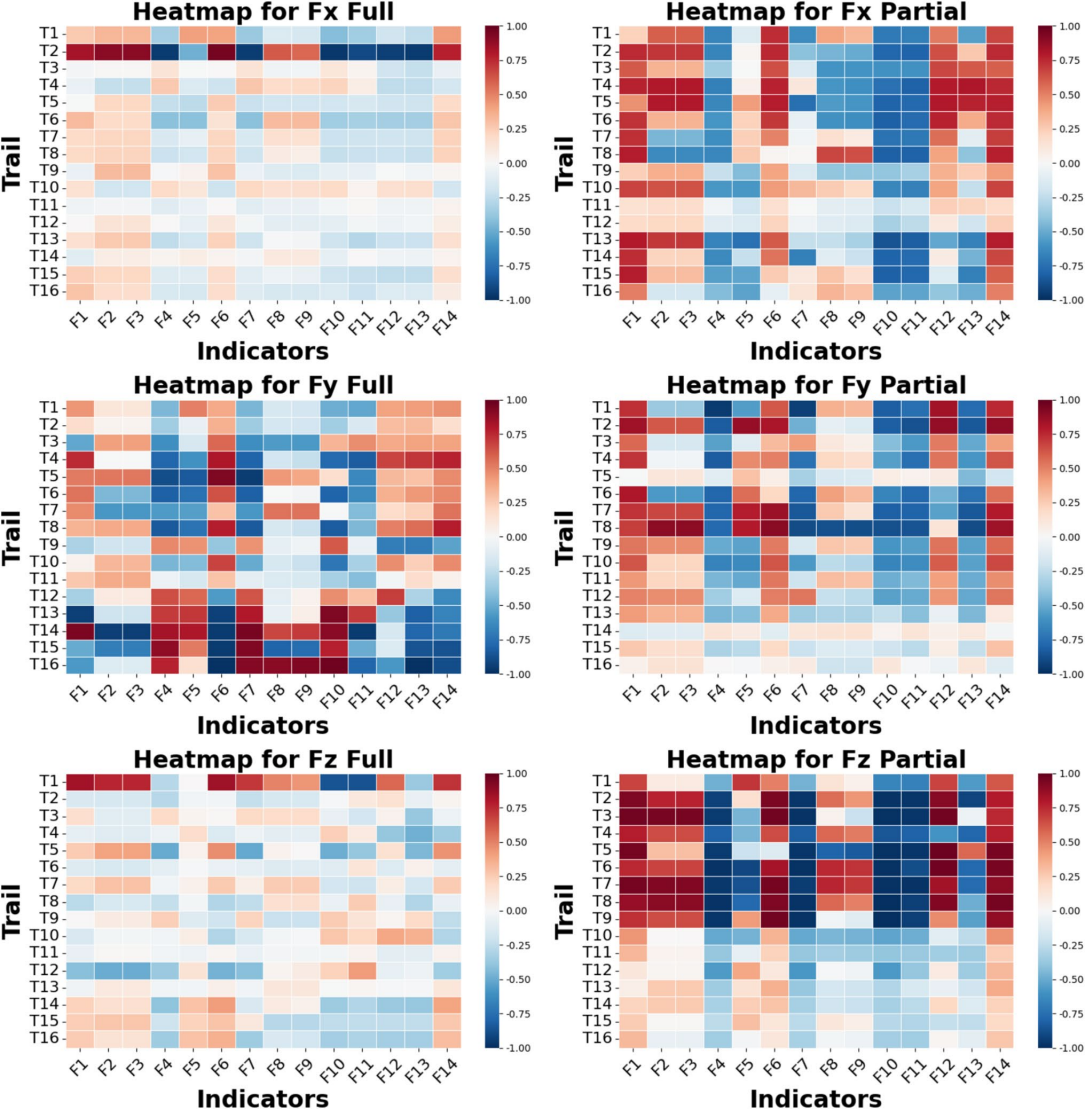
Correlation analysis heatmap

Figure 18 presents a correlation analysis of selected indicators and cutting force prediction performance, represented by ${R}^{2}$ scores, with correlation values ranging from -1.0 to 1.0. Scores close to 0 suggest minimal association between the indicators and prediction performance, while negative scores indicate an inverse relationship, and positive scores indicate a direct association. Comparing the “Full Engagement” and “Partial Engagement” stages, the predictions in the “Full Engagement” stage show a weaker correlation with the selected indicators. Analysing the correlation by system axis, the “Full Engagement” stage shows clearer correlations between most selected indicators and prediction performance in the ${F}_{y}$ axis, whereas the ${F}_{x}$ and ${F}_{z}$ axes exhibit weaker correlations, with few notable exceptions, such as **Trial 2** of the ${F}_{x}$ and **Trial 1** of the ${F}_{z}$. Like the “Full Engagement” stage, the correlation strength between selected indicators and prediction performance in the “Partial Engagement” stage varies across test cases. Interestingly, tests conducted at a spindle speed of 5000 RPM consistently show low correlation between indicators and predictive performance. Finally, among the indicators, Entropy, Smooth, RQ, and RKU generally exhibit greater correlation with predictive performance across most test cases, despite not demonstrating high sensitivity in all predictive outcomes.

## Influence of embedded TPF harmonics

The TPF and its high-order harmonics are the major components to cutting force signals. The work of this section will study the characteristics of the TPF high-order harmonics and their influence on the prediction performance with DL models.

### Embedded harmonics of TPF

The TPF and its high-order harmonics are consistently observed in most signals collected from periodically intermittent cutting processes, such as milling. In comparison to other frequency bands, the modelling of TPF and its high-order harmonics exhibits greater consistency and repeatability. This underscores the importance of investigating the impact of TPF and its harmonics on signal complexity. Initiating this exploration involves analysing the uncertainty of harmonics, which encompasses assessing the visibility of TPF harmonics, examining the uncertainty of TPF harmonics in FFT coefficients, and evaluating the repeatability of TPF harmonics. Subsequently, modelling signal complexity will proceed by employing the filtering strategy outlined in Sect. “Applied LSTM model structure and “feature function””, with a focus on harmonics.

### The proportion of the detected harmonics with different selected “active frequency” number

The visibility analysis of TPF harmonics is to summarise the proportion of the harmonic appearances while selecting different numbers of the most significant active frequency.

To achieve this goal, there are three steps: 1 sorting all extracted “active frequencies” based on their absolute values of FFT responses; 2 examining each “active frequency” with basis frequency (TPF) with 1.0 Hz as tolerance; 3 calculating the proportion of TPF harmonics in all extracted frequency.

**Fig. 19:**
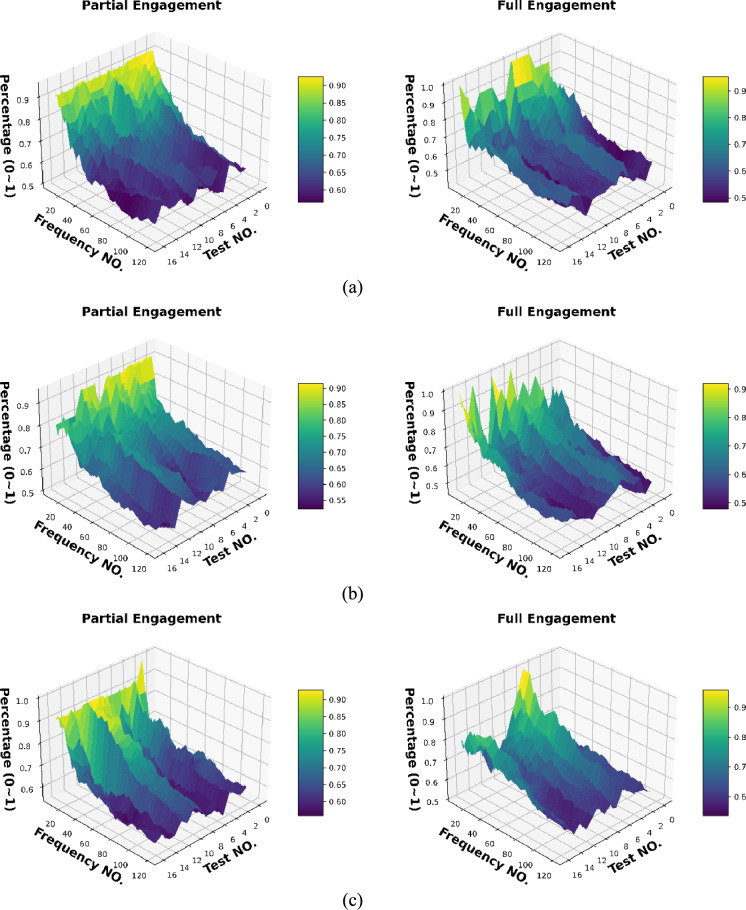
The chances of harmonics detection at **a** ${F}_{x}$, **b** ${F}_{y}$ and **c** ${F}_{z}$

Figure 19** s**hows the 3D map presents the harmonic occurrences at different preset conditions. In most cases, the number of detected harmonics becomes the majority while the lower number of total active frequencies is applied. For example, Fig. 19a shows that the harmonics detection chances in the ${F}_{x}$ of both partial engagement and full engagement stages are above 80% when the total observation number of active frequency is set to be 10. And the percentage is dropped to 50% when the total observation number of active frequency is set to be 120. Similar phenomena can be observed in ${F}_{y}$ and ${F}_{z}$ of other tests. This results clearly express the independency of the TPF harmonics detection chances, namely, it does not rely on either the setting of total observation number of active frequency, or the setting of machining parameters.

**Fig. 20:**
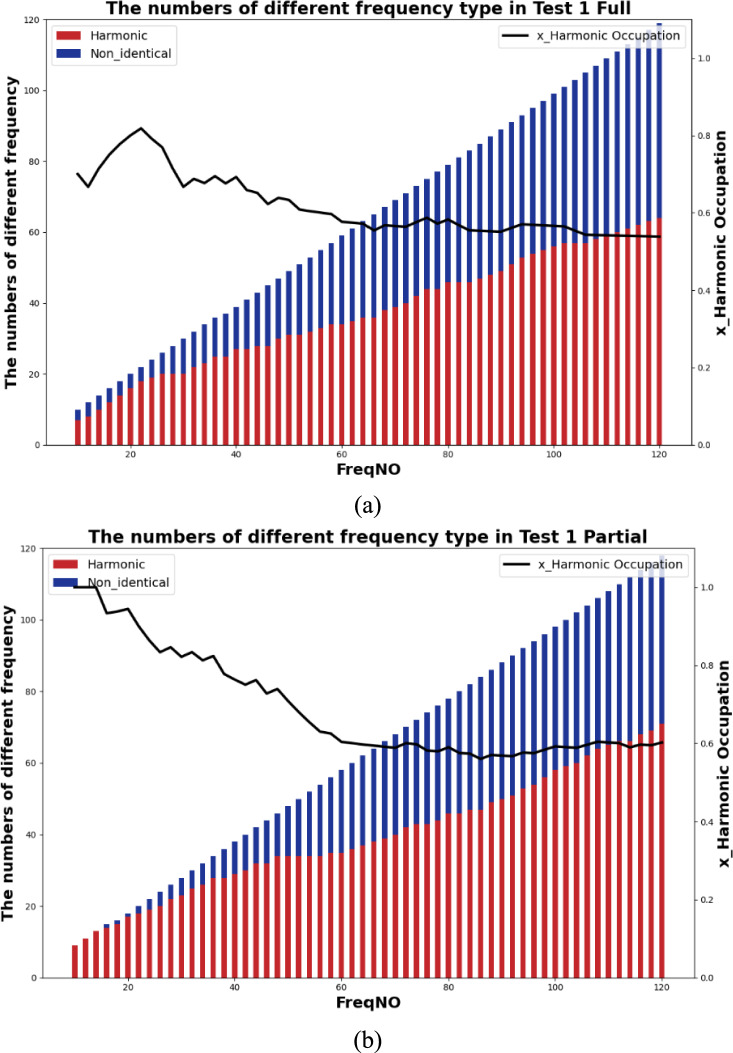
The detection proportion of the TPF harmonics: **a** the “Full Engagement” stage; **b** the “Partial Engagement” stage

Using **Test Group 1**, **Trial 1** as a case study, the analysis of the “Full Engagement” stage, depicted in Fig. 20a, reveals a discernible trend as the signal is filtered from 10 to 120 top frequencies. This filtering allows for the differentiation between harmonics and non-identical frequencies based on their occurrences. For instance, upon selecting the top 10 frequencies, 7 are identified as harmonics, while 3 are non-identical frequencies, constituting a 70% harmonic occurrence. However, as more frequencies are considered, the proportion of harmonics decreases, stabilising at around 55% with 120 top frequencies. Conversely, in the partial engagement phase illustrated in Fig. 20b, a similar trend is observed, with the proportion of harmonics declining from 100 to 55.95%. With the inclusion of additional frequencies, the prominence of non-identical frequencies increases, indicating a rise in uncertainty and signal complexity.

Therefore, several conclusions can be drawn from this analysis. Firstly, the number of TPF high-order harmonics decreases as the observed frequency target number increases. Secondly, the number of TPF high-order harmonics is independent of the observed frequency target number. Thirdly, the “Partial Engagement” stage exhibits a greater occurrence of the “decreasing” phenomenon compared to the “Full Engagement” stage. Finally, the reduction in the visibility of TPS harmonics during the “Full Engagement” stage reflects a response to changes in the machining parameters.

### Occurrence of harmonics

The previous work examined the proportion of detected TPF harmonics with varying numbers of “active frequencies.” This section will further explore the detection stability of each TPF harmonic.

**Fig. 21:**
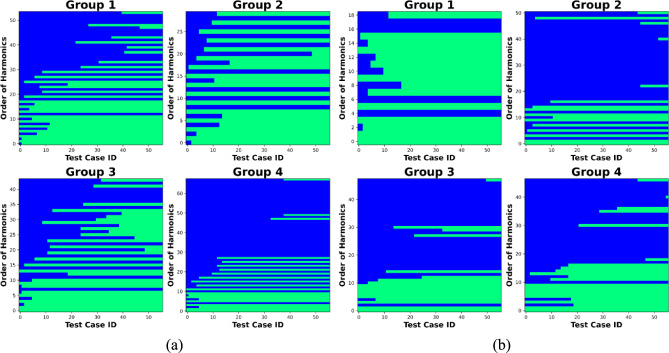
Bitmap of harmonics detection: **a** the “Partial Engagement” stage; **b** the “Full Engagement” stage

The signal analysis, particularly through the full FFT analysis, reveals the presence of numerous high-order TPF harmonics. It is crucial to understand the occurrence and signal strength of these frequencies before proceeding with further steps in performance uncertainty prediction analysis. Figure 21 presents the bitmap of TPF harmonics detection, comprising 56 observations (test cases), with the range of observed harmonic orders extending up to 60. Additionally, the analysis focused on the statistical occurrence of harmonics in relation to changes in the observed “active frequency” number. The blue areas indicate test cases where the corresponding harmonics were not detected, while the green sections highlight instances where the harmonic was detected.

In contrast to the exceptional results observed in **Test Group 2**, despite significant influence from machining environments and cutter-workpiece engagement conditions, the 1st to 10th harmonics of TPF were consistently detected across various test cases. Additionally, the number of selected “active frequencies” emerges as another factor impacting the detection of harmonics. For instance, during the “Partial Engagement” stage, the 1st to 6th harmonic orders can be reliably detected when the number of “active frequencies” exceeds 10. The influence of the preset “active frequency” number becomes more pronounced when analysing harmonic orders above the 10th. As the number of selected “active frequencies” increases, the detection of harmonics stabilises. However, as shown in Fig. 21, the influence of machining parameter settings on harmonic detection appears to be minimal.

### FFT coefficient uncertainty of the repeating harmonics

Before examining the relationship between the number of embedded TPF harmonics and prediction performance, it is important to consider the response uncertainty of each TPF harmonic as a key factor influencing the analysis of prediction performance. This consideration may help explain the causes of prediction uncertainty addressed in this paper.

**Fig. 22:**
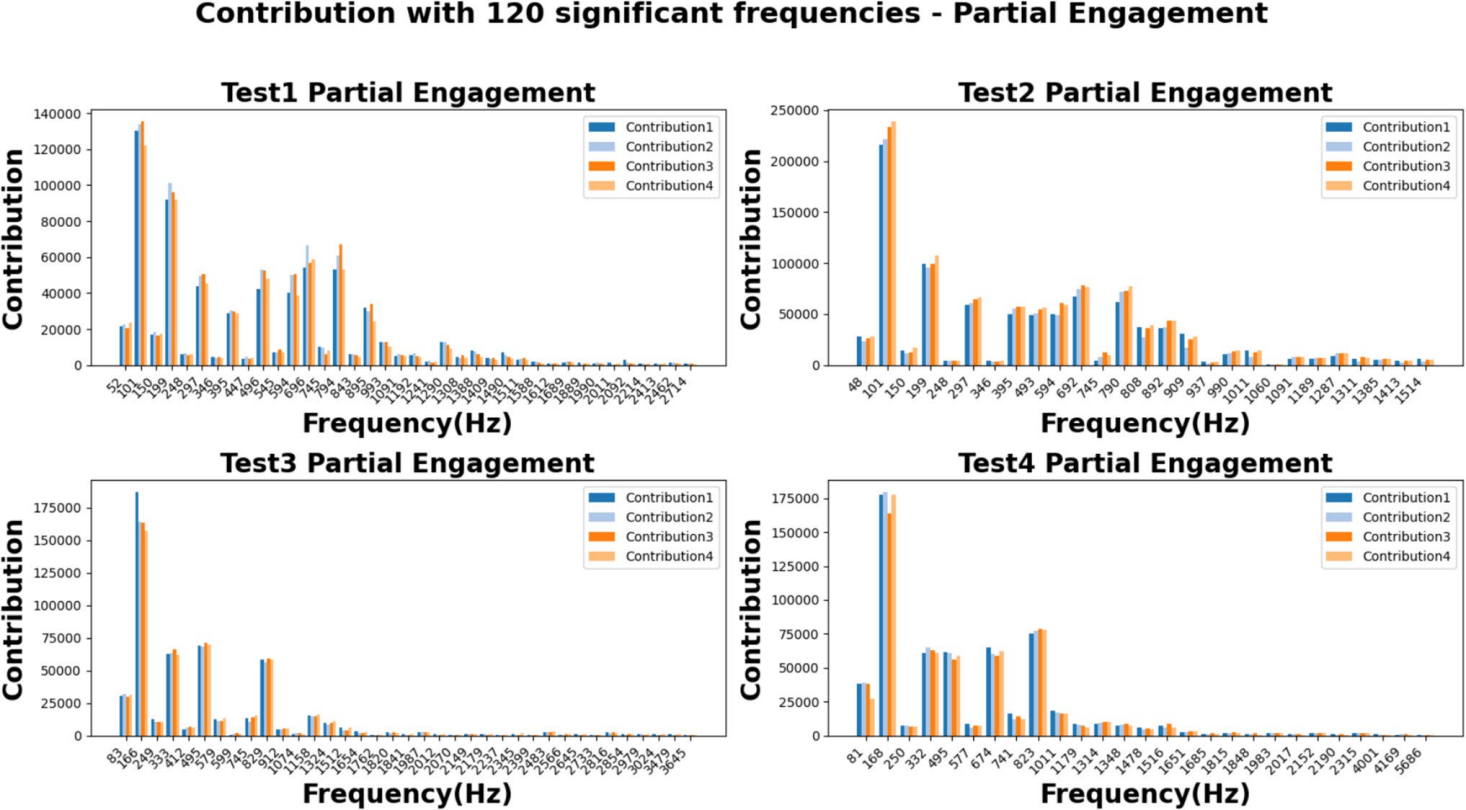
Contribution for partial engagement with top 120 significant frequencies

**Fig. 23:**
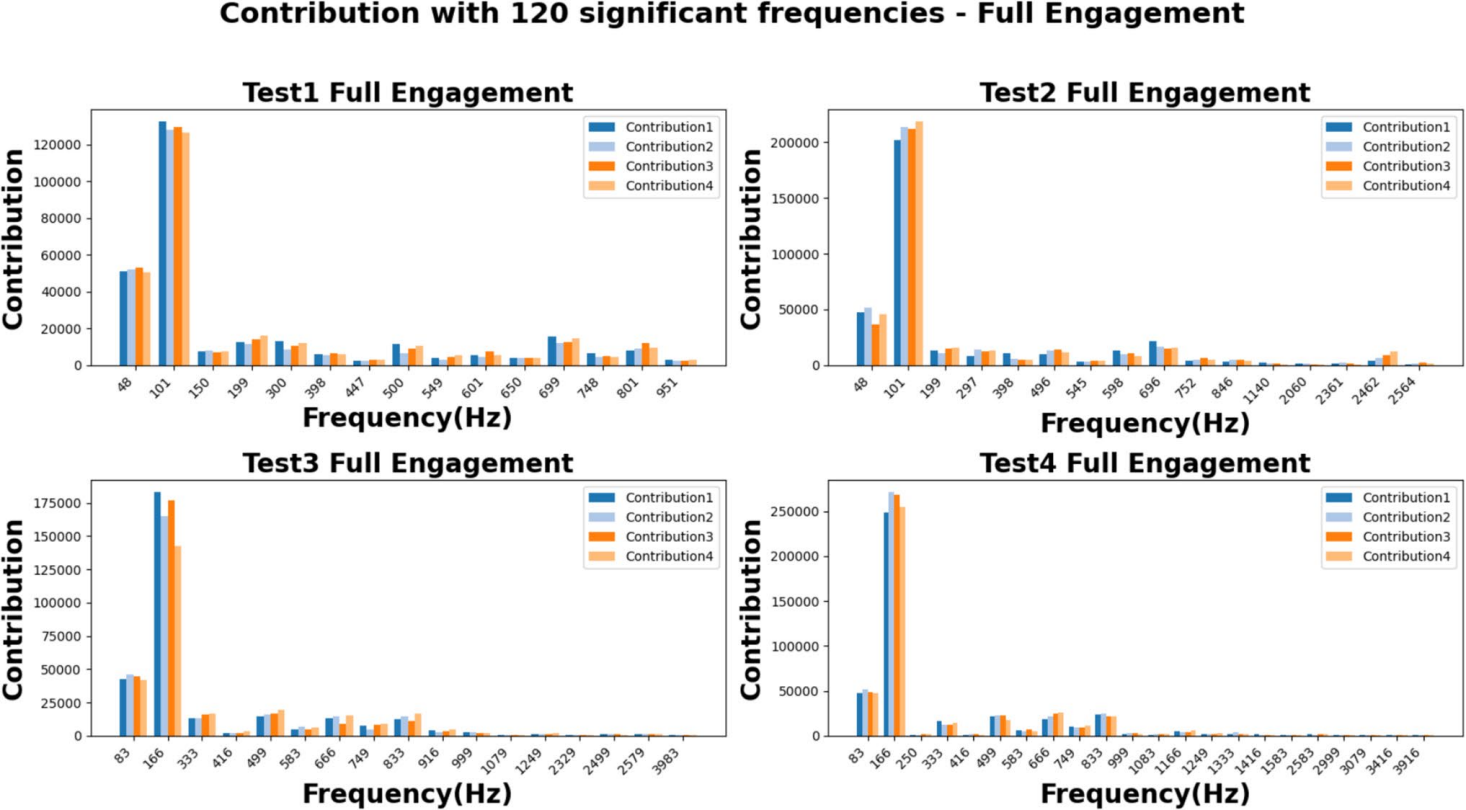
Contribution for full engagement with top 120 significant frequencies

The figures of Figs. 22 and 23 show the absolute values of FFT analysis. The corresponding values are grouped by the harmonics frequencies which were repeatedly detected in all cutting trials of each test group. In general, the harmonics with the responses higher than 20,000 at the “Partial Engagement” stage are distributed over wider frequency band than that of the “Full Engagement” stage, which only concentrated at the TPF and its first order harmonics. For example, for the **Test Group 1**, the harmonics with response higher than 20,000 are 1st, 3rd, 5th, 7th, 9th, 11th, 12th and 14th, while the statistics in Fig. 23, **Test Group 1**, only show the 1st order of TPF harmonics. Another significant difference between cutter workpiece engagement stages is the number of the TPF harmonics which can be detected in all cutting trials of each test group. This number in the “Partial Engagement” stage is higher than the “Full Engagement” stage. By considering the engagement condition of two different test scenarios, it can be concluded that the varying engagement duration enhances the response of the TPF harmonics. Nevertheless, this conclusion can also be summarised based on the previous analysis conducted in Sect. “The proportion of the detected harmonics with different selected “active frequency” number”.

In this study, the maximum differences of each observed harmonic for the “Partial Engagement” and “Full Engagement” stages are approximately 21,000 when the length of each signal sample is fixed at 20,000. The largest differences are found at the 1st order of TPF harmonics, while smaller differences are observed at higher-order harmonics, which also exhibit lower response levels compared to the low-order harmonics. For example, the differences in observed harmonics above the 10th order are less than 1,000, representing less than 5% of the corresponding value at the 1st harmonic, which is approximately 21,000.

### Harmonic predictive performance

Combining with the conclusions of previous analysis, the harmonics below order 15th have three characteristics:

- Proportion is up to 65%.
- can be stably detected, even if very few harmonics mis-detected.
- have the most significant response.

**Fig. 24:**
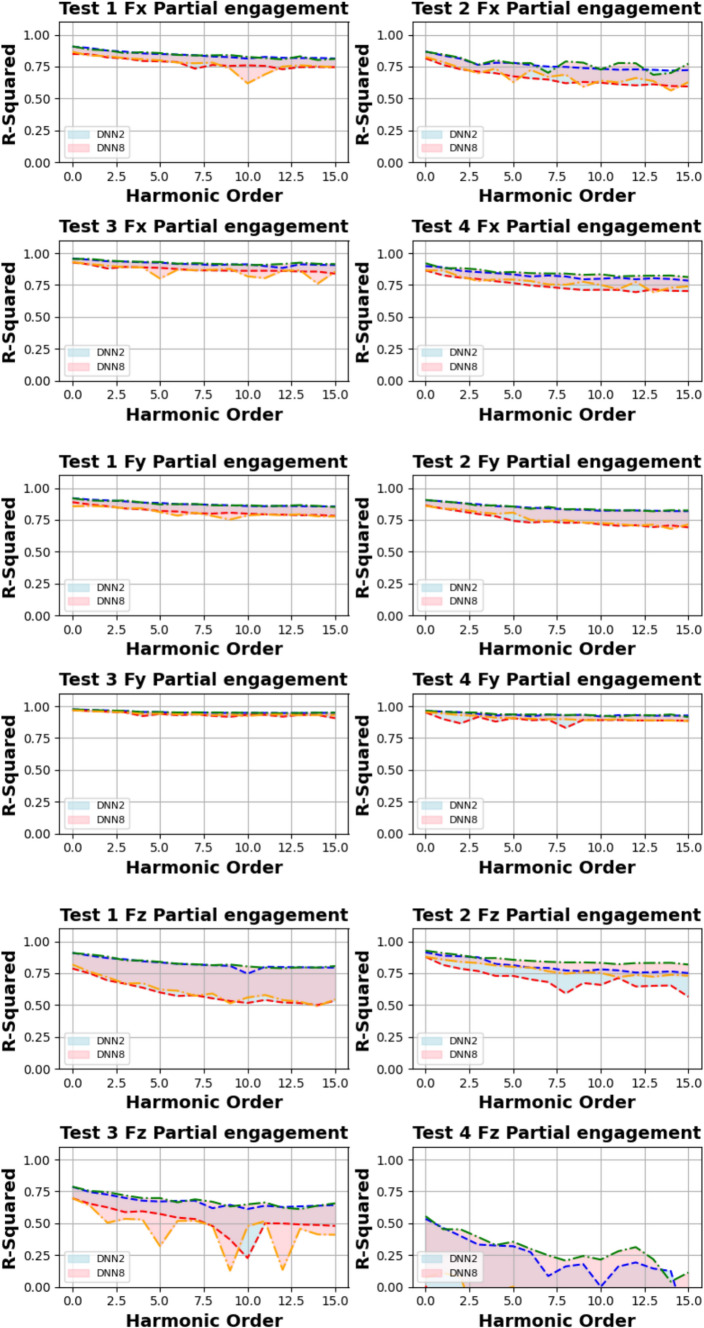
The number of embedded harmonics vs prediction performances at the “Partial Engagement” stage

**Fig. 25:**
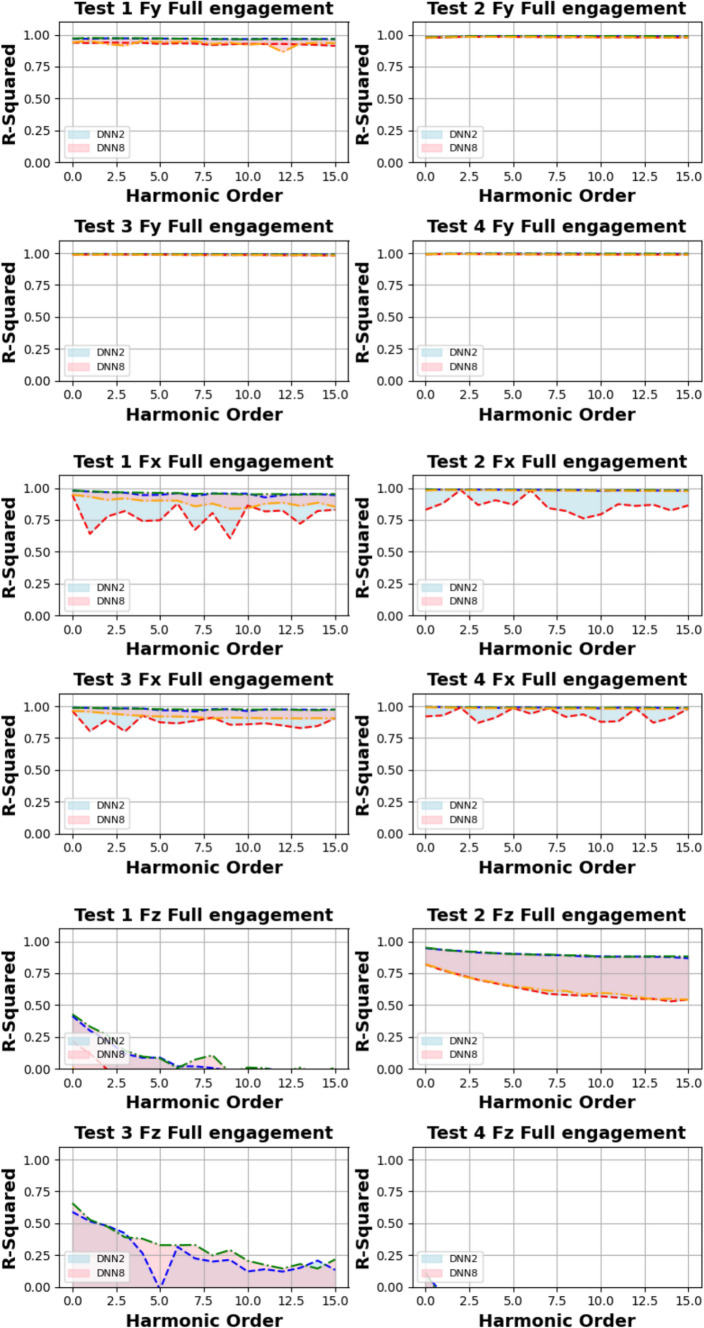
The number of embedded harmonics vs prediction performances at the “Full Engagement” stage

Based on this analysis, the subsequent prediction performance will assess the influence of changes in the number of embedded harmonics, ranging from 0 to 15. The Figs. 24 and 25 are the summary of prediction uncertainty by considering the number of embedded TPF harmonics. It should be noted that the 0 order harmonics is the spindle rotation frequency.

By comparing the results presented in Fig. 24 with those in Fig. 14, it becomes evident that the number of embedded harmonics has a more pronounced impact on the prediction of cutting force using RNN-DNN models. Most uncertainty bands for models with two “fully connected” layers overlap with those for models with eight "fully connected" layers. This suggests that increasing the number of “fully connected” layers in the RNN-DNN model has a minimal effect on prediction stability. Furthermore, this indicates that prediction stability is independent of the model structure.

From the view of descending gradients, with the increase of embedded harmonics number, the lower boundary dropping curves in some testing cases are shaper than that of upper boundary curves. This leads to the uncertainty band enlarging with the increase of embedded harmonics number. The most obvious changes are found at the ${F}_{x}$ predictions of **Test Groups 2** and **4**, ${F}_{y}$ predictions of **Test Groups 1** and **2**, ${F}_{z}$ predictions of **Test Groups 1**,** 2**,** 3**. The ${F}_{z}$ predictions of **Test Group 4** are very poor, which result in negative lower boundary.

Regarding the uncertainty band, the maximum drop in the lower and upper boundaries of ${F}_{x}$ is approximately 0.2 and 0.1, respectively. For ${F}_{y}$, the largest drops in the lower and upper boundaries are observed in **Test Group 2**, measuring around 0.1 for the lower boundary and 0.05 for the upper boundary. The most significant drops are found in the predictions of ${F}_{z}$. Excluding the exceptions in **Test Group 4**, the lower and upper boundary drops are approximately 0.25 in **Test Group 1** and 0.18 in **Test Group 3**. It is important to note that these drops only account for the ${R}^{2}$ score difference between the signal containing only TPF and the signals with all 15 harmonics and TPF.

The prediction uncertainty of ${F}_{x}$, as shown in Fig. 25, exhibits clear dependencies on the model architecture. Firstly, the overlapping areas across the four test groups are smaller than those in the corresponding test cases in Fig. 24. The overlaps are more noticeable in the comparisons between **Test Groups 1** and **3**, rather than between **Test Groups 2** and **4**. The uncertainty bands of models with eight “fully connected” layers are generally narrower than those of models with two “fully connected” layers. This indicates that models with more “fully connected” layers have a higher capacity for describing signals with varying numbers of TPF harmonics.

For the prediction of ${F}_{y}$, the results from **Test Group 1** show a slightly larger uncertainty band than the other test cases. Unlike the comparisons presented in the “Partial Engagement” stage, the width of the uncertainty bands remains relatively stable with different numbers of embedded harmonics. Additionally, the prediction uncertainty bands of the other test groups are extremely thin, indicating high prediction stability and precision. The prediction ${R}^{2}$ scores of **Test Groups 2**, **3**, and **4** are all above 0.97.

Similar to the previous analysis, the predictions of ${F}_{z}$ at the “Full Engagement” stage are highly unstable. The results not only show negative lower boundaries of the uncertainty bands but also a strong dependence on the number of embedded harmonics. The upper boundary drop-offs in **Test Groups 1** and **3** are as high as 0.4. The best predictions are observed in **Test Group 2**, where the upper and lower boundary drop-offs are around 0.05 and 0.24, respectively. The worst performance is seen in **Test Group 4**, where both the upper and lower boundary curves fall below 0.0.

### Correlation analysis of 15 indicators with prediction performance for harmonic

**Fig. 26:**
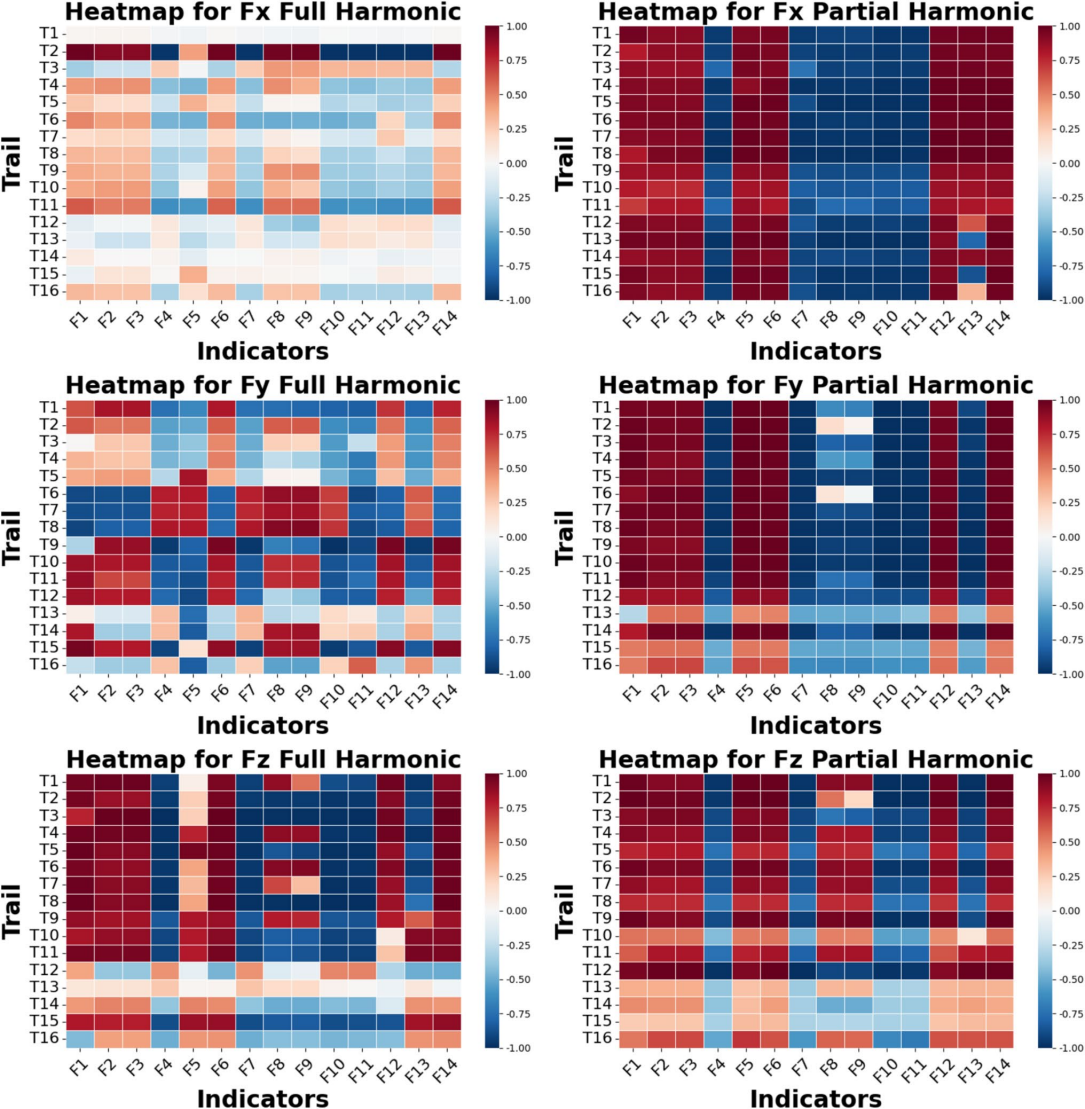
Correlation analysis heatmap

As the **Figure 26 **shows, in the ${F}_{x}$ Full heatmap, a wide range of correlations among various indicators is evident, with Relative Entropy, Cross Entropy, and Hurst showing particularly strong positive correlations with ${R}^{2}$ values. For example, in Test 1B, Relative Entropy achieves a correlation coefficient of 0.98, suggesting a significant predictive relationship. As these indicators increase, the ${R}^{2}$ values also rise, indicating their substantial role in prediction accuracy. On the other hand, indicators such as Smooth and Self Corr exhibit negative correlations, implying that higher values of these indicators are associated with reduced predictive performance. This variation in correlation strengths across different tests suggests that while certain indicators enhance model performance in specific contexts, others may detract from it, depending on the nature of the dataset and the test conditions (Fig. 26).

The ${F}_{x}$ Partial heatmap reveals similar trends, with strong positive correlations for indicators such as Entropy, Relative Entropy, Cross Entropy, Hurst, DTW, Skewness, and Noise zone. These indicators likely capture underlying system characteristics related to uncertainty and complexity, particularly highlighted by entropy-related indicators. Their increased values seem to reflect the dynamic behaviour of the system. Conversely, indicators such as Smooth, Volatility, Self-Corr, Cross-Corr, Root-Mean-Square deviation, and Maximum height of profile show strong negative correlations, indicating a limiting relationship. For instance, as Volatility increases, system smoothness tends to decrease, suggesting that a more volatile system is less predictable. These negative correlations highlight the inverse relationship between system complexity and smoothness, further emphasising the significance of volatility in prediction accuracy.

The ${F}_{y}$ Full heatmap exhibits more mixed correlation patterns. Positive correlations are observed for indicators such as Entropy and Hurst, while indicators like Self-Corr show negative correlations with ${R}^{2}$ values. The significant negative correlation for Self-Corr suggests that it does not contribute positively to model accuracy in this context. Similar patterns are observed in the ${F}_{y}$ Partial heatmap, where Entropy, Relative Entropy, Cross Entropy, Hurst, DTW, Skewness, and Noise zone continue to demonstrate strong positive correlations. Indicators such as Smooth, Volatility, Self-Corr, and Root-Mean-Square deviation again show negative correlations, implying that system complexity is inversely related to smoothness and volatility. These patterns are also evident in the ${F}_{z}$ Full and Partial heatmaps, where positive correlations among Cross Entropy, and Hurst suggest their key role in capturing system uncertainty and complexity. Negative correlations for Smooth and Volatility highlight their tendency to reduce predictive accuracy by complicating the modelling process, especially under conditions of increased volatility.

The key features of this correlation analysis are the identification of both positive and negative relationships between various indicators and ${R}^{2}$ values across multiple datasets. Indicators such as Entropy, Relative Entropy, Cross Entropy, and Hurst consistently show strong positive correlations, indicating their effectiveness in capturing system complexity and uncertainty. These indicators highlight a direct relationship with predictive performance, as higher values generally correspond to improved accuracy. Conversely, indicators like Smooth and Volatility demonstrate strong negative correlations, suggesting they may introduce noise or unpredictability, thus reducing model performance. As for Harmonics, the correlation presents more significant performance compared with the signal of the general frequency. In partial engagement, Entropy, relative entropy, cross entropy, Hurst DTW, Skewness (RSK) and Noisezone present strong positive correlation, while Smooth, Volatility, Root-Mean-Square (RQ), Maximum height of profile (RZ) present strong negative correlation. As for full engagement, it is difficult to give a unique indicator to present the correlation performance, but for most of Test group, entropy could present positive correlation and Maximum height of profile (RZ) present negative correlation. Both strongly positive and negative correlations provide valuable insights into signal complexity and prediction effectiveness. These indicators, whether positively or negatively correlated, can effectively represent signal complexity and serve as key predictors of model performance.

## Discussion

### Instability of ${F}_{z}$

Although ${F}_{z}$ is typically considered less significant in cutting force models and is often excluded from tool wear prediction due to its instability and noise, its inclusion in this study remains justified for several reasons. First, frequency distribution analysis (Fig. 10) reveals that the tooth passing frequency (TPF) and its harmonics can be distinctly identified in the ${F}_{z}$ component with minimal interference from noise. Second, the analysis of time-series factors (Figs. 8 and 9) demonstrates that ${F}_{z}$ exhibits consistent monotonicity features, reinforcing its relevance in signal interpretation. Finally, prediction performance analysis (Fig. 24) indicates that ${F}_{z}$ is particularly sensitive to the number of integrated TPF harmonics, highlighting its potential to enhance model accuracy and responsiveness. Despite its inherent instability, these factors suggest that ${F}_{z}$ contributes valuable information to the analysis, supporting its retention in this study.

### Classification performance analysis with different level of signal complexity

As a supervised learning technique, the purpose of classification for cutting force process is to classify the different statuses of data including noise, partial engagement and full engagement. As for the classification method, CNN is employed with three 1D convolution layers with ReLU function. In general, cutting signals exhibit distinct characteristics and high recognition performance. For single-axis analysis, when fewer signals are introduced, the recognition performance of ${F}_{x}$ and ${F}_{z}$ is low due to incomplete feature recognition. As more signals are added, the increasing accumulation of signal-specific features enhances recognition performance. Among the axes, Fy demonstrates the highest recognition performance, reaching 100%. As more frequencies of signal are added, the signal complexity will increase, thus, the characteristics of signal will be significant, which will be helpful to complete the classification with a high accuracy.

**Fig. 27:**
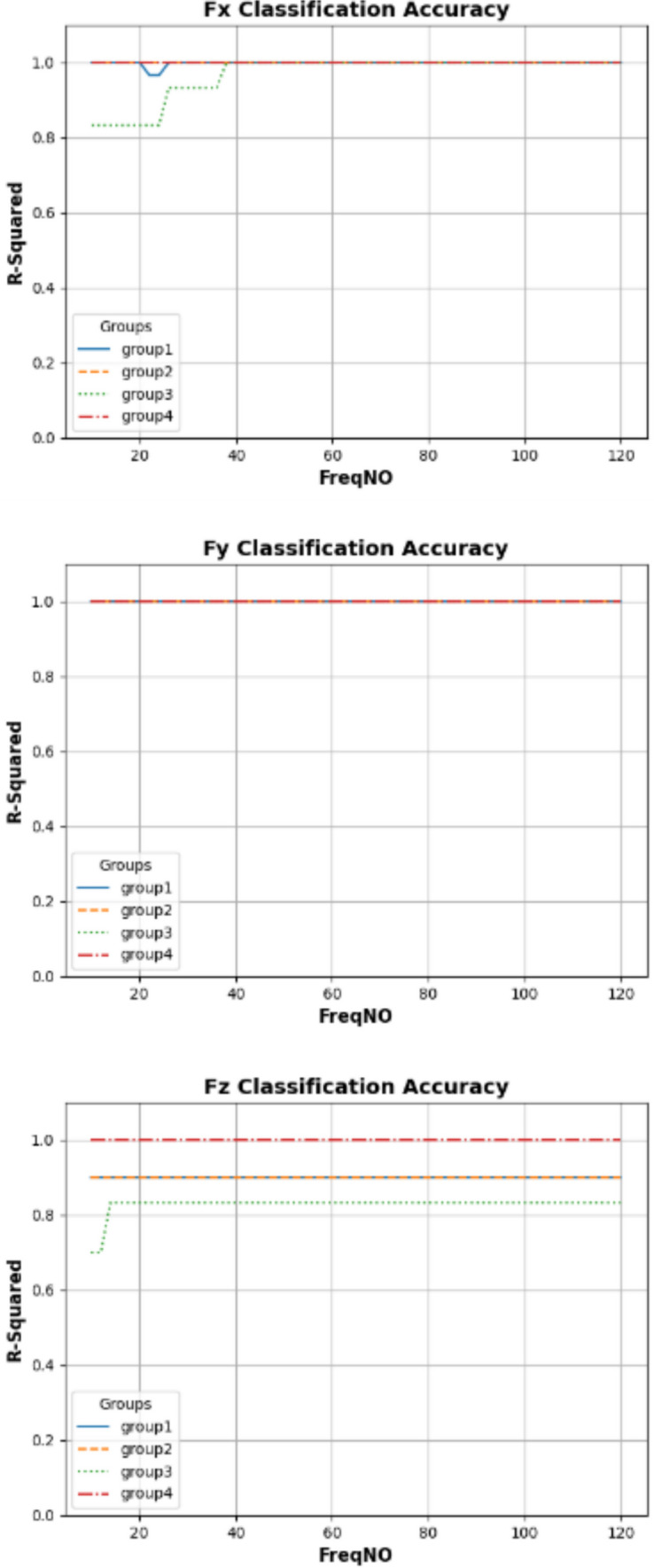
Classification accuracy of three axes

From the **Figure 27**, as for ${F}_{x}$, all the accuracies of classification reach 83.3% above. After keeping more than 38 frequencies of data, the accuracy of classification could reach 100%, which means noise, partial engagement and full engagement could be classified clearly. The characters at various stages exhibit distinct pattern variations clearly. As for ${F}_{y}$, the accuracies of classification are 100%. As for ${F}_{z}$, Test 1 and Test 2 have 90% of accuracy, Test 3 has lower accuracy of 70%, and keep 83.3% from 14 to 120 frequencies of data. Test 4 has 100% accuracy. The result of classification shows that uncertainty information during different cutting force stages could not affect the pattern of signals seriously. It is straightforward to classify the three patterns (Fig. 27).

The utilisation of convolutional neural networks (CNN) for classification in the cutting force process demonstrates promising results, particularly in accurately distinguishing between noise, partial engagement and full engagement states. The employment of three 1D convolution layers with ReLU activation functions contributes to robust classification performance. Notably, for $Fx$, achieving accuracies of 83.3% or higher indicates the effectiveness of the classification method in capturing distinct pattern variations across different stages of the cutting force process. Moreover, attaining 100% accuracy in classification after retaining more than 38 frequencies of data underscores the clear delineation of noise, partial engagement, and full engagement statuses.

Similarly, for $\mathrm{Fy}$, achieving 100% accuracy in classification further validates the efficacy of the classification approach in accurately identifying different signal patterns. However, for ${F}_{z}$, variations in accuracy across different tests highlight potential differences in signal characteristics or machining conditions. Despite lower accuracies for some tests, such as test 3 with 70% accuracy, retaining a sufficient number of frequencies (83.3% from 14 to 120 frequencies) ensures robust classification performance.

Overall, the findings indicate that the inherent uncertainty across various cutting force stages minimally influences signal patterns, thereby enabling clear classification of noise, partial engagement, and full engagement states. This underscores the reliability and consistency of the classification method in capturing critical signal characteristics essential for effective process monitoring and control. Consequently, these results offer valuable insights for refining classification techniques in machining processes, ultimately enhancing efficiency and productivity in manufacturing operations.

## Conclusion

This study explores the performance of DL model-based signal processing technology under different machining conditions, with a focus on understanding how signal complexity influences model reliability and effectiveness. The significance of this work lies in its contribution to establishing credibility measures for DL model architectures in machining diagnostics. In industrial applications, the design or selection of DL models can rely on signal complexity analysis by using the 14 selected factors to ensure process quality and efficiency.

The analysis progresses to the concept of active frequencies, which are the dominant components significantly contributing to the signal. Digital filtering techniques, particularly a Butterworth filter, were utilized to manage these active frequencies, leading to enhanced prediction accuracy. The investigation revealed that the complexity of cutting force signals is heavily influenced by the number of retained frequencies, with significant contributions arising from those below 1000 Hz. The introduction of 14 features indicators provided a robust framework for quantifying signal complexity and correlating it with predictive performance.

The applied LSTM-DNN in this work generated promising predictions with the help of TPF feature functions. The findings indicate that predictions during the “Partial Engagement” stages were more stable compared to those during the “Full Engagement” stage. Specifically, ${F}_{z}$ predictions revealed significant variability and susceptibility to noise, particularly in Test 2, which demonstrated the weakest performance for ${F}_{x}$ and ${F}_{y}$ with *R*^2^ values dropping below 0.7 due to limited cutting depth. Furthermore, the analysis of DL model performance in this study demonstrates that the LSTM-DNN architecture is well-suited to the milling process, where cutting forces exhibit dynamic variation. This contrasts with turning and drilling, where forces remain relatively stable. Consequently, the validated LSTM-DNN models can be reasonably expected to achieve high accuracy in force prediction across other cutting methods.

Additionally, the study underscored the importance of selecting appropriate machining parameters. Tests operating at higher RPMs (5000) and feed rates (1500 mm/min) consistently yielded improved prediction accuracy, with the best *R*^2^ for ${F}_{x}$ observed in Test 4 at 0.9530, demonstrating that optimal combinations of cutting parameters significantly enhance LSTM prediction accuracy during partial engagement. Conversely, the performance in full engagement showed stable *R*^2^ values but highlighted a distinct variability between different engagement states, reinforcing the need for tailored parameter selection to optimize predictive capabilities.

The investigation into TPF and its high-order harmonics illustrated their critical role in shaping the characteristics of cutting force signals. As the number of observed active frequencies increased, the detection of TPF harmonics decreased, highlighting the necessity to balance signal complexity with effective model training. The correlation analysis identified key indicators, such as Cross Entropy, which showed strong positive correlations with predictive accuracy, emphasizing their importance in model refinement. Negative impacts were noted from indicators such as Smoothness, which adversely affected predictive performance.

In terms of classification, the CNN utilized in this study achieved notable success in distinguishing between noise, partial engagement, and full engagement states. The classification accuracy improved as more signal frequencies were incorporated, with ${F}_{y}$ achieving a perfect classification rate. However, variability in ${F}_{z}$ performance indicated the influence of machining conditions, necessitating further investigation into the effects of signal characteristics on classification robustness.

Overall, the study affirms the robustness of the methodologies employed in capturing and analysing cutting force signals, facilitating effective process monitoring and control in manufacturing operations. The insights derived contribute significantly to the refinement of predictive models and enhance understanding and optimization of machining processes. Future research should focus on exploring additional feature extraction methods and model architectures, aiming to improve both classification performance and prediction accuracy, especially in more complex or uncertain scenarios. This research lays the groundwork for advancements in predictive maintenance strategies and intelligent manufacturing systems, ultimately promoting enhanced efficiency and productivity within the industry.

Future research can be extended to various machining processes and different material applications. This would lead to two key objectives: (i) the development of autonomous DL models optimised for enhanced computational efficiency and deployment, and (ii) AI-driven process inference to improve diagnostic capabilities in machining operations.

## Data Availability

The data supporting the findings of this study are not publicly available due to confidentiality agreements stemming from business collaborations. However, they may be made available by the authors upon reasonable request for restricted purposes. Access to the data will require approval from the Centre for Precision Technology at the University of Huddersfield and may be subject to additional conditions.

## Abbreviations

AI: Artificial intelligent
AE: Acoustic emission optimization
ANN-POA: ANN pigeon optimization algorithm
BPPE: Best peak prediction error
CNC: Computer numeric control
CNN: Convolution neural network
CWE: Cutter-workpiece-engagement
DIV: Divergence
DL: Deep learning
DNN: Deep neural network
DOC: Depth of cut
FEM: Finite element method
FFT: Fast fourier transformation
JC: Johnson-Cook
LSTM: Long-short-term-memory
$$lb$$: Look back
MAE: Mean absolute error
ML: Machine learning
MSE: Mean squared error
PPM: Post-processing model
${R}^{2}$: Coefficient of determination
RNN: Recurrent neural networks
ReLU: Rectified linear unit
RQ: Root-mean -square deviation
RZ: Maximum height of profile
RSK: Skewness
RKU: Kurtosis
SAS: Semi-analytical solution
Self-Corr: Self-correlation
TL: Transfer learning
TPF: Tooth passing frequency

## List of symbols

$$CC$$: Accumulated contribution
${f}_{t},{f}_{d}$: Feed per second, feed per min
$${f}_{g,n}$$: Feature function
${f}_{low}$: Low frequency band
${f}_{high}$: High frequency band
${F}_{x},{F}_{y},{F}_{z}$: Cutting force in workpiece coordinates
$N$: Flute number of cutters
$$n$$: Spindle speed
${r}_{tool}$: Milling tool radius
$\omega$: Radians per second
$$t$$: Cutting time
${t}_{n}$: Cutting time stamp, n = $\mathrm{1,2},\dots$
${x}_{t}$: Instantaneous position of cutter nose in feed direction

## References

[CR1] Abebe, R., & Gopal, M. (2023). Exploring the effects of vibration on surface roughness during CNC face milling on aluminium 6061–T6 using sound chatter. *Material Today: Proceeding,**90*, 43.

[CR2] Ali, Y. M. B. (2023). Adversarial attacks on deep learning networks in image classification based on smell bees optimization algorithm, future generation computer. *System,**140*, 185–195.

[CR3] Al-Selwi, S. M., Hassan, M. F., Abdulkadir, S. J., Muneer, A., Sumiea, E. H., Alqushaibi, A., & Ragab, M. G. (2024). RNN-LSTM: From applications to modelling techniques and beyond-Systematic review. *Journal of King Saud University—Computer and Information Sciences,**36*, 102068.

[CR4] Alzugaray-Franz, R., Diez, E., Villaverde, M., & Vizan, A. (2024). Indirect measurement of process parameters in peripheral end milling based on acoustic emission signals. *Measurements,**234*, 1114801.

[CR5] Axinte, D. A., Belluco, W., & Chiffre, L. D. (2001). Evaluation of cutting force uncertainty components in turning. *International Journal of Machine Tools & Manufacture,**41*, 719–730.

[CR6] Baldán, F. J., & Benítez, J. M. (2023). Complexity meaxures and features for time series classification. *Expert System with Applications,**213*, 119227.

[CR7] Bhattacharyya, A., Schueller, J. K., Mann, B. P., Schmitz, T. L., & Gomez, M. (2021). Uncertainty propagation through an empirical model of cutting forces in end milling. *Journal of Manufacturing Science and Engineering,**143*, 071002–071011.

[CR8] Bhattacharyya, P., Sengupta, D., & Mukhopadhyay, S. (2007). Cutting force-based real-time estimation of tool wear in face milling using a combination of signal processing techniques. *Mechanical Systems and Signal Processing,**21*, 2665–2683.

[CR9] Budach, L., Feuerpfeil, M., Ihde, N., Nathansen, A., Noack, N., Patzlaff, H., Naumann, F., Harmouch, H. (2022). The effects of data quality on machine learning performance, arXiv:2207.14529.

[CR10] Chauhan, S., Trehan, R., & Singh, R. P. (2024). Classification of surface roughness for CNC face milling of Inconel 625 superalloy utilizing cutting force signal features with SVM and ANN. *Material Today: Proceedings,**113*, 9.

[CR11] Chen, Y., Ling, G., Song, X. X., & Tu, W. H. (2023). Characterizing the statistical complexity of nonlinear time series via ordinal pattern transition networks. *Physica a: Statistical Mechanics and Its Applications,**618*, 128670.

[CR12] Dai, W., Yoshigoe, K., Parsley, W. (2018). Improving data quality through deep learning and statistical models, 10.1007/978-3-319-54978-1_66.

[CR13] Das, A., & Bajpai, V. (2023). Turning insert with lubricating passage along the normal plane for minimization of friction, cutting force and tool wear. *Journal of Manufacturing Processes,**101*, 141–155.

[CR14] Das, B., Roy, S., Rai, R. N., & Saha, S. C. (2016). Study on machinability of in situ Al-4.5%Cu-TiC metal matrix composite-surface finish, cutting force prediction using ANN. *CIRP Journal of Manufacturing Science and Technology,**12*, 67–78.

[CR15] Ding, P. F., Huang, X. Z., Miao, X. L., Li, S. J., & Liu, H. Z. (2023). Dynamic stability simulation of micro-milling under the condition of multi-parameter uncertainty. *Probabilistic Engineering Mechanics,**74*, 103499.

[CR16] Ducroux, E., Fromentin, G., Viprey, F., Prat, D., & D’Acunto, A. (2021). New mechanistic cutting force model for milling additive manufactured Inconel 718 considering effects of tool wear evolution and actual tool geometry. *Journal of Manufacturing Processes,**64*, 67–80.

[CR17] Gauder, D., Biehler, M., Gölz, J., Schulze, V., & Lanza, G. (2022). In-process acoustic pore detection in milling using deep learning. *CIRP Journal of Manufacturing Science and Technology,**37*, 125–133.

[CR18] Gong, Y., Liu, G. Z., Xue, Y. Z., Li, R., & Meng, L. Z. (2023). A survey on dataset quality in machine learning. *Information and Software Technology,**162*, 107268.

[CR19] Gözü, E., & Karpat, Y. (2017). Uncertainty analysis of force coefficients during micromilling of titanium alloy. *International Journal of Advanced Manufacturing Technology,**93*, 839–855.

[CR20] Hajdu, D., & Bachrathy, D. (2023). Active vibration control for milling operations including frequency response function uncertainties. *Procedia CIRP,**117*, 181–186.

[CR21] Heitz, T., Bachrathy, D., He, N., Chen, N., & Stepan, G. (2023). Optimization of cutting force fitting model by Fast Fourier Transformation in milling. *Journal of Manufacturing Process,**99*, 121–137.

[CR22] Kakade, S., Vijayaraghavan, L., & Krishnamurthy, R. (1994). In-process tool wear and chip-form monitoring in face milling operation using acoustic emission. *Journal of Materials Processing Technology,**44*, 207–214.

[CR23] Kim, J., Jung, W., An, J., Oh, H. J., & Park, J. H. (2023). Self-optimization of training dataset improves forecasting of cyanobacterial bloom by machine learning. *Science of the Total Environment,**866*, 161398. 36621510 10.1016/j.scitotenv.2023.161398

[CR24] Kouguchi, J., & Yoshioka, H. (2024). Monitoring method of cutting forces and vibrations by using frequency separation of acceleration sensor signals during milling process with small ball end mills. *Precision Engineering,**85*, 337–356.

[CR25] Li, G., Li, C., Wen, C., & Ding, S. (2018). Investigation and modelling of flank wear process of different PCD tools in cutting titanium alloy Ti6Al4V. *International Journal of Advanced Manufacturing Technology,**95*, 719–733.

[CR26] Li, G., Munir, K., Wen, C., Li, Y., & Ding, S. (2020). Machinablility of titanium matrix composites (TMC) reinforced with multi-walled carbon nanotubes. *Journal of Manufacturing Processes,**56*, 131–146.

[CR27] Li, G., Rahim, M. Z., Ding, S., & Sun, S. (2016). Performance and wear analysis of polycrystalline diamond (PCD) tools manufactured with different methods in turning titanium alloy Ti-6Al-4V. *International Journal of Advanced Manufacturing Technology,**85*, 825–841.

[CR28] Li, G., Xu, W., Jin, X., Liu, L., Ding, S., & Li, C. (2023a). The machinability of stainless steel 316 L fabricated by selective laser melting: Typical cutting responses, white layer and evolution of chip morphology. *Journal of Materials Processing Technology,**315*, 117926.

[CR29] Li, M., Chen, Y. J., Tan, M. B., Yang, X. J., & Xiao, Z. (2023b). Surface integrity and acoustic emission characteristics during slot milling 3D carbon/carbon composites using superabrasive diamond grinding point. *Diamond & Related Materials,**137*, 110166.

[CR30] Li, X. J., Li, Y., Feng, Z. W., Wang, Z. X., & Pan, Q. (2023c). ATS-O2A: a state-based adversarial attack strategy on deep reinforcement learning. *Computers & Security,**129*, 103259.

[CR31] Liu, C., Huang, Z. P., Huang, S. F., He, Y., Yang, Z. D., & Tuo, J. B. (2023b). Surface roughness prediction in ball screw whirlwind milling considering elastic-plastic deformation caused by cutting force: Modelling and verification. *Measurement,**220*, 113365.

[CR32] Liu, M., Xie, H., Pan, W., Ding, S., & Li, G. (2023a). Prediction of cutting force via machine learning: state of the art, challenges and potentials. *Intelligent Manufacturing,**1*, 1–62.

[CR33] Liu, Y., Li, T., Liu, K., & Zhang, Y. M. (2016). Chatter reliability prediction of turning process system with uncertainties. *Mechanical Systems and Signal Processing,**66–67*, 232–247.

[CR34] Ma, Y. C., Tao, L., Rong, Z., Tang, K. H., Huang, Z. Q., & Chen, K. (2023). Research on expression method of PDC bit wear uncertainty and influence of lateral force. *Geoenergy Science and Engineering,**255*, 211700.

[CR35] Moreira, L. C., Li, W., Lu, X., & Fitzpatrick, M. E. (2019). Supervision controller for real-time surface quality assurance in CNC machining using artificial intelligence. *Computers & Industrial Engineering,**127*, 158–168.

[CR36] Ng, K. W., Huang, Y. F., Koo, C. H., Chong, K. L., El-Shafie, A., & Ahmed, A. N. (2023). A review of hybrid deep learning applications for streamflow forecasting. *Journal of Hydrology,**625*, 130141.

[CR37] No, T., Gomez, M., Karandikar, J., Heigel, J., Copenhaver, R., & Schmitz, T. (2021b). Propagation of Johnson-Cook flow stress model uncertainty to milling force uncertainty using finite element analysis and time domain simulation. *Procedia Manufacturing,**53*, 223–235.

[CR38] No, T., Gomez, M., & Schmitz, T. (2021a). Contributions of scanning metrology uncertainty to milling force prediction. *Procedia Manufacturing,**53*, 213–222.

[CR39] Paletta, Q., Terrén-Serrano, G., Nie, Y., Li, B. H., Bieker, J., Zhang, W., Dubus, L., Dev, S., & Feng, C. (2023). Advances in solar forecasting: Computer vision with deep learning. *Advances in Applied Energy,**11*, 100150.

[CR40] Rajesh, A., Prabhuswamy, M., & Krishnasamy, S. (2022). Smart manufacturing through machine learning: a review, perspective, and future directions to the machining industry. *Journal of Engineering*. 10.1063/5.0131827

[CR41] Sakthivel, N. R., Cherian, J., Nair, B. B., Abburu Sahasransu, L. N. V., Aratipamula, P., & Gupta, S. A. (2024). An acoustic dataset for surface roughness estimation in milling process. *Data in Brief,**57*, 111108. 39633973 10.1016/j.dib.2024.111108 PMC11615534

[CR42] Sessions, V., Valtorta, M. (2006). The effects of data quality on machine learning algorithms, MIT International Conference

[CR43] Singh, K. K., & Singh, R. (2020). Process mechanics based uncertainty modelling for cutting force prediction in high speed micromilling of Ti6Al4V. *Procedia Manufacturing,**48*, 273–282.

[CR44] Soori, M., Arezoo, B., & Dastres, R. (2023). Machine learning and artificial intelligence in CNC machine tools. A review. *Sustainable Manufacturing and Service Economics,**2*, 100009.

[CR45] Tansel, I., Trujillo, M., Nedbouyan, A., Velez, C., Bao, W. Y., Arkan, T. T., & Tansel, B. (1998). Micro-end-milling-III. Wear estimation and tool breakage detection using acoustic emission signals. *International Journal of Machine Tools & Manufacture,**38*, 1449–1466.

[CR46] Twarddowski, P., Tabaszewski, M., Pikula, M. W., & Czyryca, A. F. (2021). Identification of tool wear using acoustic emission signal and machine learning methods. *Precision Engineering,**72*, 738–744.

[CR47] Uhlmann, E., & Holznagel, T. (2022). Acoustic emission-based process monitoring in the milling of carbo fibre-reinforced plastics. *CIRP Journal of Manufacturing Science and Technology,**37*, 464–476.

[CR48] Wang, K., Chen, Z. C., Dang, X. L., Fan, X., Han, X. M., Chen, C. M., Ding, W. P., Yiu, S. M., & Weng, J. (2023). Uncovering hidden vulnerabilities in convolutional neural networks through graph-based adversarial robustness evaluation. *Pattern Recognition,**143*, 109745.

[CR49] Wang, S. Q., He, C. L., Li, J. G., & Wang, J. (2021). Vibration-free surface in the milling of a thin-walled cavity part using a corn starch suspension. *Journal of Material Processing Technology,**290*, 116980.

[CR50] Waqas, M., & Humphries, U. W. (2024). A critical review of RNN and LSTM variants in hydrological time series predictions. *MethodsX,**13*, 102946. 39324077 10.1016/j.mex.2024.102946 PMC11422155

[CR51] Wazirali, R., Yaghoubi, E., Abujazar, M. S. S., Ahmad, R., & Vakili, A. H. (2023). State-of-the-art review on energy and load forecasting in microgrids using artificial neural networks, machine learning, and deep learning techniques. *Electric Power Systems Research,**225*, 109792.

[CR52] Wei, J. F., Yao, L. Y., & Meng, Q. G. (2023). Self-adaptive logit balancing for deep neural network robustness: Defend and detection of adversarial attacks. *Neurocomputing,**531*, 180–194.

[CR53] Wojciechowski, S., Matuszak, M., Powałka, B., Madajewski, M., Maruda, R. W., & Krolczyk, G. M. (2019). Prediction of cutting forces during micro end milling considering chip thickness accumulation. *International Journal of Machine Tools & Manufacture,**147*, 103466.

[CR54] Wu, G., Li, G., Pan, W., Wang, X., & Ding, S. (2020). A prediction model for the milling of thin-wall parts considering thermal-mechanical coupling and tool wear. *International Journal of Advanced Manufacturing Technology,**107*, 4645–4659.

[CR55] Wu, X., Zhang, C., Li, Y., Huang, W. Z., Zeng, K., Shen, J. Y., & Zhu, L. F. (2023). Researches on tool wear progress in milling-grinding based on the cutting force and acceleration signal. *Measurement,**218*, 113234.

[CR56] Xiang, W. Z., Su, H., Liu, C., Guo, Y. D., & Zheng, S. B. (2023). Improving the robustness of adversarial attacks using an affine-invariant gradient estimator. *Computer Vision and Image Understanding,**299*, 103647.

[CR57] Xu, K., Li, Y. G., Zhang, J. C., & Chen, G. X. (2021). ForceNet: An offline cutting force prediction model based on neuro-physical learning approach. *Journal of Manufacturing Systems,**61*, 1–15.

[CR58] Xu, W., Wang, C., Long, Y., Li, C., Li, G., & Ding, S. (2024). The influence of deformation affected region on microstructure and mechanical property of 316L fabricated by hybrid additive-subtractive manufacturing. *Journal of Manufacturing Processes,**117*, 154–169.

[CR59] Zhang, P. F., Gao, D., Lu, Y., Ma, Z. F., Wang, X. R., & Song, X. (2022). Cutting tool wear monitoring based on a smart toolholder with embedded force and vibration sensors and an improved residual network. *Measurement,**199*, 111520.

[CR60] Zhang, X., Wang, X., Zhang, P., Chen, K., & Cao, F. (2024). Fast extraction of cutter-workpiece engagement for milling force prediction in multi-axis machining. *Measurement,**231*, 114490.

[CR61] Zhuang, K., Gao, J. Q., Ye, T., & Dai, X. (2022). Effect of cutting edge radius on cutting force and surface roughness in machining of Ti-6Al-4V. *Procedia CIRP,**108*, 571–576.

